# A clinical evaluation of the performance of five commercial artificial intelligence contouring systems for radiotherapy

**DOI:** 10.3389/fonc.2023.1213068

**Published:** 2023-08-04

**Authors:** Paul J. Doolan, Stefanie Charalambous, Yiannis Roussakis, Agnes Leczynski, Mary Peratikou, Melka Benjamin, Konstantinos Ferentinos, Iosif Strouthos, Constantinos Zamboglou, Efstratios Karagiannis

**Affiliations:** ^1^ Department of Medical Physics, German Oncology Center, Limassol, Cyprus; ^2^ Department of Radiation Oncology, German Oncology Center, Limassol, Cyprus; ^3^ School of Medicine, European University Cyprus, Nicosia, Cyprus; ^4^ Department of Radiation Oncology, Medical Center – University of Freiberg, Freiberg, Germany

**Keywords:** AI, contouring, radiotherapy, breast, head and neck, lung, prostate

## Abstract

**Purpose/objective(s):**

Auto-segmentation with artificial intelligence (AI) offers an opportunity to reduce inter- and intra-observer variability in contouring, to improve the quality of contours, as well as to reduce the time taken to conduct this manual task. In this work we benchmark the AI auto-segmentation contours produced by five commercial vendors against a common dataset.

**Methods and materials:**

The organ at risk (OAR) contours generated by five commercial AI auto-segmentation solutions (Mirada (Mir), MVision (MV), Radformation (Rad), RayStation (Ray) and TheraPanacea (Ther)) were compared to manually-drawn expert contours from 20 breast, 20 head and neck, 20 lung and 20 prostate patients. Comparisons were made using geometric similarity metrics including volumetric and surface Dice similarity coefficient (vDSC and sDSC), Hausdorff distance (HD) and Added Path Length (APL). To assess the time saved, the time taken to manually draw the expert contours, as well as the time to correct the AI contours, were recorded.

**Results:**

There are differences in the number of CT contours offered by each AI auto-segmentation solution at the time of the study (Mir 99; MV 143; Rad 83; Ray 67; Ther 86), with all offering contours of some lymph node levels as well as OARs. Averaged across all structures, the median vDSCs were good for all systems and compared favorably with existing literature: Mir 0.82; MV 0.88; Rad 0.86; Ray 0.87; Ther 0.88. All systems offer substantial time savings, ranging between: breast 14-20 mins; head and neck 74-93 mins; lung 20-26 mins; prostate 35-42 mins. The time saved, averaged across all structures, was similar for all systems: Mir 39.8 mins; MV 43.6 mins; Rad 36.6 min; Ray 43.2 mins; Ther 45.2 mins.

**Conclusions:**

All five commercial AI auto-segmentation solutions evaluated in this work offer high quality contours in significantly reduced time compared to manual contouring, and could be used to render the radiotherapy workflow more efficient and standardized.

## 1 Introduction

In radiation treatment planning, contouring (also called segmentation or delineation) of organs-at-risk (OARs) and target volumes is a vital part of the radiation therapy treatment chain (1, 2). These contours are usually generated manually by a radiation oncologist or radiotherapy technologist (RTT), but the process is subjective and thus dependent on the user’s experience (3–5) as well as the imaging modalities available to inform the process (6). Contour inconsistencies lead to variations in the quality of the plan and, ultimately, patient outcomes, and it has been reported that worse disease control and increased toxicity can be attributed to poor contouring (7–9). Despite a vast array of contouring guidelines (10, 11), inter-observer, and even intra-observer, variations remain an issue. Auto-segmentation has been long proposed as a solution to reduce such variations and to reduce the time spent on this manual task (12–14), with artificial intelligence and deep-learning algorithms showing great promise (15–17).

Time savings are always considered valuable as they allow the radiation oncologist to spend more time on other activities, such as patient contact or peer review. However, it is vital that artificial intelligence (AI) contours are critically reviewed prior to their use in the clinic. The AI solutions are generated based on a specific training dataset, which can vary between hundreds to thousands of patients. Individual patient anatomies can easily differ from these model training datasets, however, with anatomical variations, surgical removal of tissue, presence of non-biological materials and different imaging protocols leading to minor (boundary miss) or major (missing slices) segmentation errors (18). As such, it is recommended that every output of an auto-segmentation application should be reviewed, corrected if needed, and approved prior to clinical use (18). If the time to correct to the AI contours becomes comparable to the time required to draw the contours manually, the user should question whether there is any use for the AI model.

A plethora of commercial deep learning segmentation solutions is available for a center that wishes to standardize and speed up their contour generation. While there have been several benchmarking studies demonstrating the high quality of contours generated, the authors are not aware of any publications explicitly comparing the contours produced by different AI-segmentation commercial vendors to a common dataset. Other similar works include the AAPM Grand Challenges at the 2017 (19) and 2019 (20) meetings (on thoracic CT auto-segmentation and head and neck MRI segmentations, respectively), however competition entries did not have to be an approved commercial product. In our work we compare the contours produced by five commercial AI auto-segmentation solutions, Mirada, MVision, Radformation, RayStation and TheraPanacea, for a set of 80 patients (20 breast, 20 head and neck, 20 lung, 20 prostate).

In evaluating the performance of these auto-segmentation solutions, the goals of using such a system should be considered. As stated previously, auto-segmentation solutions offer the opportunity for more consistent contours and a reduction in contouring time (21–24). As such, in this study assessments are made using geometric scoring, which assess the variability of contours, and time-based scoring, which allows for an understanding of the impact on clinical workflow. It is hoped that such an assessment will provide additional information to the procurement process for radiation therapy centers and will encourage further improvements to the standards of auto-segmentation solutions.

## 2 Material and methods

### 2.1 Clinical contours

A total of 80 patients were assessed that were previously treated in our clinic: 20 from each of four anatomical sites (breast, head and neck, lung, and prostate). Selection was random, based on the date of treatment but it was confirmed that the sample size was sufficiently large that it provided a robust dataset to ensure sufficient stress-testing of the algorithm, which has been shown to be an important consideration when testing auto-contouring solutions (25). For instance, the breast cases were balanced between 11 right and 10 left (one case was bilateral), 5 thoracic wall, 5 involving supra-clavicular fossa, 3 involving the axilla and 1 treating the internal mammary node. The demographic also ensured adequate testing of the solutions, with a median (range) age of 64 years (39-87), weight 71 kg (46-138), height 1.62 m (1.52-1.68) and BMI 26.1 (19.9-50.7).

Contours generated from each of the AI solutions were compared to expert contours drawn by three Radiation Oncologists, all with at least ten years’ contouring experience, following protocols and guidelines (26–28) as detailed in **Table 1**. Contours were peer reviewed after one Radiation Oncologist contoured all the structures for a given anatomical site. These manual contours were drawn using ProSoma v4.1 (MedCom GmbH, Darmstadt, Germany) using a brush tool, with interpolation between slices (which is routine clinical practice). In editing the AI contours, a brush tool was again used to push in/out the contour lines, together with an eraser when required. The same Radiation Oncologist that drew the original contours also corrected the AI contours, blinded from the original contours, with a gap of at least six weeks from the original contouring.

**Table 1:** Contouring guidelines followed by our clinicians when drawing the expert contours.

Anatomical Region	Organ	Guidelines	Mir	MV	Rad	Ray	Ther
Breast	Breast	RTOG atlas 2008	(29, 30)	(29)	RTOG atlas 2008	(11)	(29)
	Heart	RTOG atlas 2008	(31)	(32)	(32)	(11)	(29)
	Humerus	RTOG atlas 2008		Internal guidelines			(29)
	Liver	RTOG atlas 2008			Upper abdominal RTOG Atlas 2013	(11)	(29)
	Lung	RTOG atlas 2008	(32)	(32)	(32)	(32)	(29)
	Oesophagus	RTOG atlas 2008	(27, 32)			(11)	(29)
	SpinalCanal	RTOG atlas 2008	Internal guidelines	(32)	RTOG 1016 2011	(11)	(29)
Head and neck	Brain	(28)	(27)	(27)	(27)		33
	Brainstem	(27)	(27)	(28)	(27)	(28)	33
	Chiasm	(28)	(27)	(28)	(27)		33
	Eye	(27)	(27)	(28)	(27)	(28)	33
	Hyoid	(27)					
	Hypophysis	(27)	(27)	(27)	(27)	(28)	33
	InnerEar	(27)	Internal guidelines				
	LacrimalGl	(27)	(27)	(27)	(27)	(28)	
	Larynx	(27)	34	(27)	RTOG 1016 2011	(27)	(29)
	Lens	(27)	(27)	(28)	(27)	(28)	33
	Mandible	(27)	(27)	(27)	(27)	(27)	33
	OpticNerve	(28)	(27)	(27)	(27)	(27)	
	ParotidGl	(27)	(27)	(27)	(27)	(35)	
	SpinalCord	(27)	(27)	(27)	RTOG 1016 2011	(27)	
	Sternocleido	(27)					
	SubmandGl	(27)	(27)	(27)	(27)	(35)	33
	Trachea	(27)	Internal guidelines	Internal guidelines		(11)	32
Lung	Heart	RTOG atlas 2008	31	(32)			
	Liver	RTOG atlas 2008		(36)	(27)		
	Lung	RTOG atlas 2008	32	(32)			32
	Oesophagus	RTOG atlas 2008	(27)	(36)	(32)	(11)	32
	SpinalCanal	RTOG atlas 2008	(27)	(32)	RTOG 1016 2011		32
Prostate	Bladder	(26)	(26)	(26)	(26)	(26)	
	Bowels	(26)	(26)	(26)	(26)		
	CaudaEquina	(26)				(11)	
	FemoralHead	(26)	(26)	(26)	(26)	(26)	
	PenileRoot	(26)	(26)	37	(26)		
	Prostate	(26)	(26)	37	(26)	(26)	
	Rectum	(26)	(26)	37	(26)	(26)	
	Sigmoid	(26)		(26)			
	SeminalVes	(26)	(26)	(26)	(26)		

Taking into account laterality (counting individually for left and right) and excluding the patient external, the number of contours for each patient were: nine for breast; 17 for head and neck; six for lung; 10 for prostate. Any contours excluded from the study are indicated with the * in **Table 1**, with the motivation for the exclusion given in Section 2.4.

### 2.2 AI contouring systems

In this study the following five systems were tested: DLCExpert v2.6.4.47181 from Mirada Medical (Oxford, UK); MVision v1.2.1 (Helsinki, Finland); AutoContour v1.0.25.0 from Radformation (New York, USA), Deep Learning Segmentation within the RayStation Treatment Planning System v12.0.0.932 from RaySearch (Stockholm, Sweden); and Annotate v1.10.0 from TheraPanacea (Paris, France) (henceforth referred to as Mir, MV, Rad, Ray and Ther, respectively). In such a crowded, competitive, environment all systems undergo constant development and updates. This work was conducted in April 2022 and thus corresponds to the status of each system at that time.

All systems separate the implementation into individual models for different anatomical locations. However, most systems offer the option to either define custom models, in which the structures to be included are manually selected, or to run any model on any image dataset. Therefore, it is not logical to list the structures included in each model, but rather all the possible structures that can be generated with each system (across all a system’s models). These are listed in **Supplementary Table 1**.

### 2.3 Contour evaluation

Each AI contour was compared to its corresponding expert contour, which was manually-drawn by a Radiation Oncologist. A multitude of similarity metrics are available when quantitatively comparing the similarities of two structures, as detailed by Taha and Hanbury (38). However, as detailed by Sherer et al., no single metric can effectively measure the quality of a contour (39). In this work we use a number of geometric metrics, each of which have advantages and disadvantages, as well as make an assessment of the time to correct the AI contours.

#### 2.3.1 Geometric metrics

The volumetric Dice Similarity Coefficient (vDSC) is a metric that measures the amount of overlap of two contours and is commonly used in studies comparing different segmentations of the same organ. Simple to compute, it is defined as the union of two volumes (A and B) normalized to the mean of the two volumes, as shown in equation 1:


(1) $$vDSC=\frac{2|A\cap B|}{\left|A|+|B\right|}.$$

The vDSC has a score between 0 and 1, with a value of 1 when the two contours exactly overlap. While it has been utilized frequently, it has been shown that it does not correlate with the clinical quality of the contours or the time to adjust them (40), it does not differentiate between systematic and random errors, and it is not sensitive to complex boundaries. Additionally, the vDSC is biased to give higher scores for larger volumes.

In radiotherapy planning, contouring is performed on a slice by slice basis so the surface Dice similarity coefficient (sDSC), proposed by Nikolov et al. (41), is a more appropriate metric. It assesses the agreement of two contours rather than two volumes and is defined as the union of two contours (S_1_ and S_2_) normalized to the mean surface of the two contours within a tolerance parameter *τ*, as shown in equation 2:


(2) $$sDSC=\frac{\left|S_{1}\cap B_{2,\tau }\right|+\left|S_{2}\cap B_{1,\tau }\right|}{\left|S_{1}\right|+\left|S_{2}\right|}.$$

It has been shown that sDSC has a good correlation with time required to edit contours (42, 43), however the tolerance parameter *τ*, which represents inter-observer variations in segmentations, must be set appropriately. Nikolov et al. (41) determined values of *τ* between 0.97-2.93 mm for organs in the head and neck. In this work the voxelized version of the contours (i.e. binary masks) is analyzed, not the original (subvoxel) contours. As we wanted to analyze all edits no additional tolerance was added, so the tolerance is, at most, equal to the voxel size.

The distance between boundaries can be assessed using the Hausdorff distance (HD) (40, 44–46). Computed as the maximum nearest neighbor Euclidean distance between two volumes, it is sensitive to boundary errors (14, 47). However, as stated in the introduction, one of the primary motivators for the use of auto-segmentation tools within radiotherapy is to save time and it has been shown that HD does not correlate with the time required to edit contours because it does not account for the proportion of edits needed (40, 48). Nonetheless, HD is a common metric that is well understood by most readers, and it has been suggested that because volumetric overlap and distance metrics are not highly correlated they are potentially complementary (14). For these reasons it is included in this study.

Additionally, the added path length (APL), proposed by Vaassen et al. (42) and defined as the total length of contour that must be corrected to make the two contours overlap, was determined. It has been shown that this has good correlation with the time required for contouring (42). The APL accounts for the number of slices an organ encompasses and is not normalized by volume, which makes it unbiased for volumetrically small but lengthy organs such as the esophagus, which are often subject to poor visualization (39). The APL assumes lines are drawn to edit contours, however if a brush tool is used it may be more appropriate to consider the added volume, such as the false negative volume proposed by Kiser et al. (43). In our work we computed APL with no additional tolerance so that all edits could be assessed, so the tolerance is, at most, equal to the voxel size.

Visual representation of the four above metrics can be found in Vaassen et al. (42). As stated previously, each of the above metrics (vDSC, sDSC, HD and APL) have advantages and disadvantages. Potentially a composite of the above metrics may assist in determining contour quality, but inherently there is a limitation in that none of the metrics are able to distinguish where the variation is located. In this work all four of the above geometric indices have been determined for each contour, on each patient, for each AI contouring system (using Open Reggui (https://www.openreggui.org/), but it is necessary to also consider non-geometric assessments, which are explained in the proceeding sections.

#### 2.3.2 Timing comparisons

Although it has been shown that auto-segmentation tools offer time-saving compared to manual contouring (22, 40, 49–51), manual reviews are necessary and corrections are often required. While the sDSC and APL geometric indices can be used as surrogates of the time-saving offered by AI auto-segmentation systems, in this work we manually recorded the absolute times to draw the expert contours as well as the times required to correct the AI contours.

The times required by each system to generate the AI contours are of the order of a few minutes, but these are not relevant in a clinical setting because these processes can be configured to operate in the background of clinical operation. With the exception of RayStation’s deep learning segmentation, which must be processed within the treatment planning solution (TPS), the other systems offer a solution in which the images from the CT are automatically intercepted by the AI auto-segmentation system, appropriate contours (dependent on the anatomical site) are automatically generated, and the CT images and RT structure sets are automatically pushed to a desired destination for review and for the radiation oncologist to add their target volumes. As such, the time to generate the AI contours can be considered to be zero or insignificant, which is the assumption made in this work. However, to ensure the AI system meets clinical expectations and to ensure consistency and accuracy, it is critical that all AI-generated contours are reviewed. Thus, for AI systems the relevant time to consider is the time to correct the AI-generated contours so that they are considered appropriate for clinical use. In this work such correction times were recorded, for a randomly selected subset of the patients (three) in each anatomical site and on each system.

There is a potential for bias because each system does not contour the same structures (e.g. a given system may only contour a well-defined structure like the lung and thus the overall correction time will be shorter). Therefore, if a system did not generate a contour that the institution does, it was assigned the average correction time from the other systems that *did* generate that contour. To allow computation of the time saving, only the structures contoured routinely by our institution were corrected. The absolute and relative time savings (in minutes and as percentage, respectively) were computed.

### 2.4 Excluded and combined structures

To ensure a fair comparison, some structures had to be excluded from the study, even if they are routinely contoured in the clinic. The larynx can be contoured following a variety of guidelines [RTOG 1016, (52, 53)], which may explain poor results reported in literature [e.g. vDSC=0.28 was reported by Guo et al. (54)]. We found that the different systems followed different guidelines, so for fairness larynx was excluded throughout our comparison. As detailed in **Table 1**, other structures follow different guidelines but the impact was much less dramatic so they were still compared. The hyoid, inner ear and sternocleidomastoid muscles were excluded due to lack of AI structures.

**Supplementary Figure 1** shows the different contouring approaches to the optic nerves and optic chiasm – it is not clear where the boundary between the optic nerves and optic chiasm should be drawn. We approached this by grouping the optic chiasm and left and right optic nerves into a common structure called the ‘optic pathway’. Most systems contour the optic chiasm and optic nerves according to the guidelines of Brouwer et al. (27) (based on CT), but in our clinic we routinely use MRI so follow the guidelines of Scoccianti et al. (28). MV provides contours that follow the latter guidelines, so those structures were assessed. Assessment of the optic pathway rather than individual structures should eliminate discrepancies between CT and MRI based contouring. Ray was excluded from this combined structure because, although it did contour the optic nerves, it did not contour the optic chiasm.

## 3 Results

**Figure 1:**
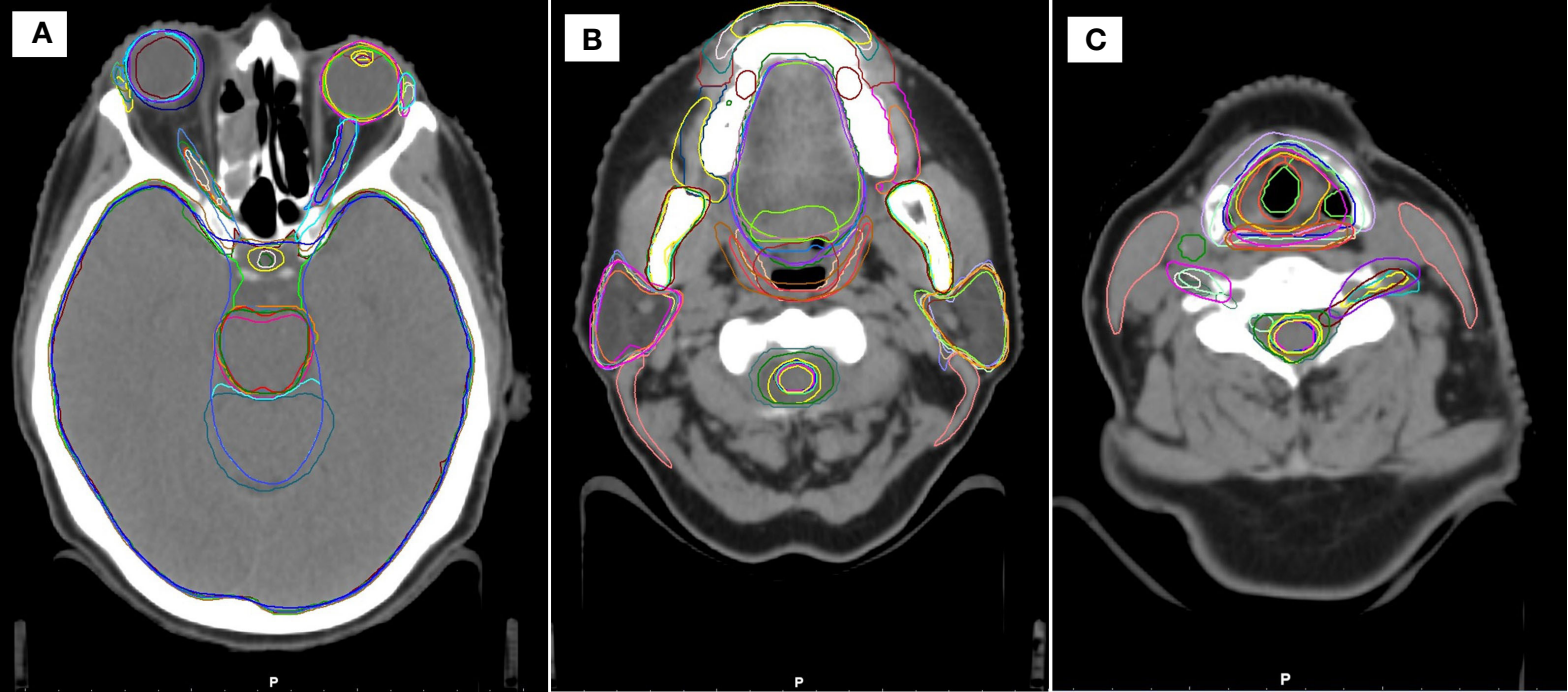
Example head and neck contours in the study, showing similarities and differences between the different systems at three cranial-caudal heights **(A–C)**.

As can be seen in **Supplementary Table 1**, the total number of structures (including laterality) for each AI contouring software are: Mir 99; MV 142; Rad 83; Ray 67; Ther 86. In this work only a proportion of these structures were tested (10 for breast, 19 for head and neck, 6 for lung, 10 for prostate) as these correspond to structures routinely contoured in our clinic. As an illustration, example structures for a head and neck case, are shown in **Figure 1**. Some structures are very similar (eyes, mandible), whereas other show significant differences (brainstem, oral cavity, larynx).

### 3.1 Similarity comparison metrics

**Figure 2:**
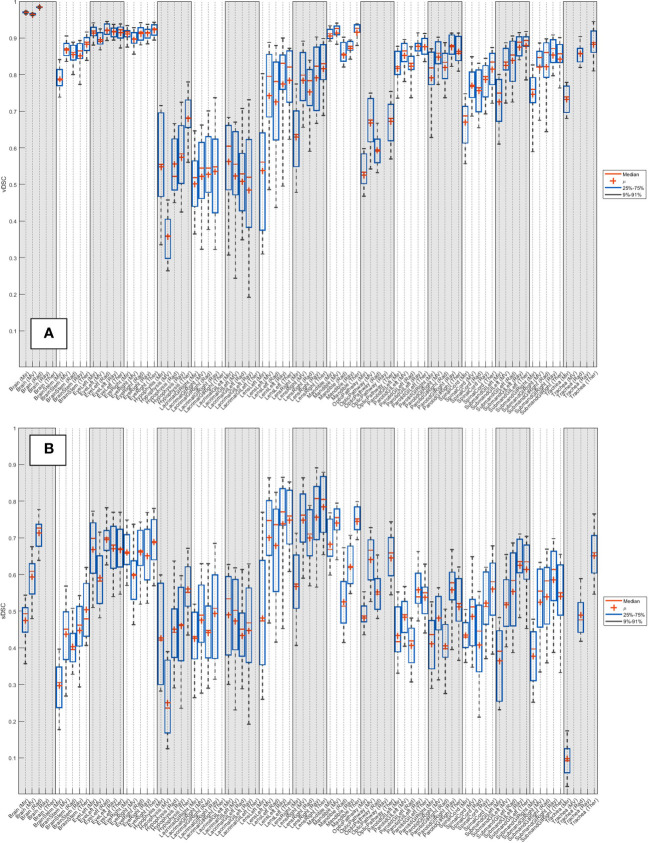
Distribution of **(A)** vDSC and **(B)** sDSC (with no added tolerance) across the 20 patients for each system, for the 19 organs at risk routinely contoured for head and neck.

The median vDSC and sDSC indices for all systems across the 20 patients are detailed in **Supplementary Table 2** (breast), **Supplementary Table 4** (head and neck), **Supplementary Table 6** (lung) and **Supplementary Table 8** (prostate). The distribution of results across the 20 patients is shown as boxplots in **Supplementary Figure 2** (breast), **Figure 2** (head and neck), **Supplementary Figure 4** (lung) and **Supplementary Figure 6** (prostate).

The corrected segmentations were found to agree better with the expert contours than the original AI segmentations, with geometric similarity coefficients increasing. For instance, the corrected breast OAR vDSCs demonstrated an absolute increase of 0.01/0.02/0.06/0.01/0.02 for Mir/MV/Rad/Ray/Ther respectively.

**Figure 3:**
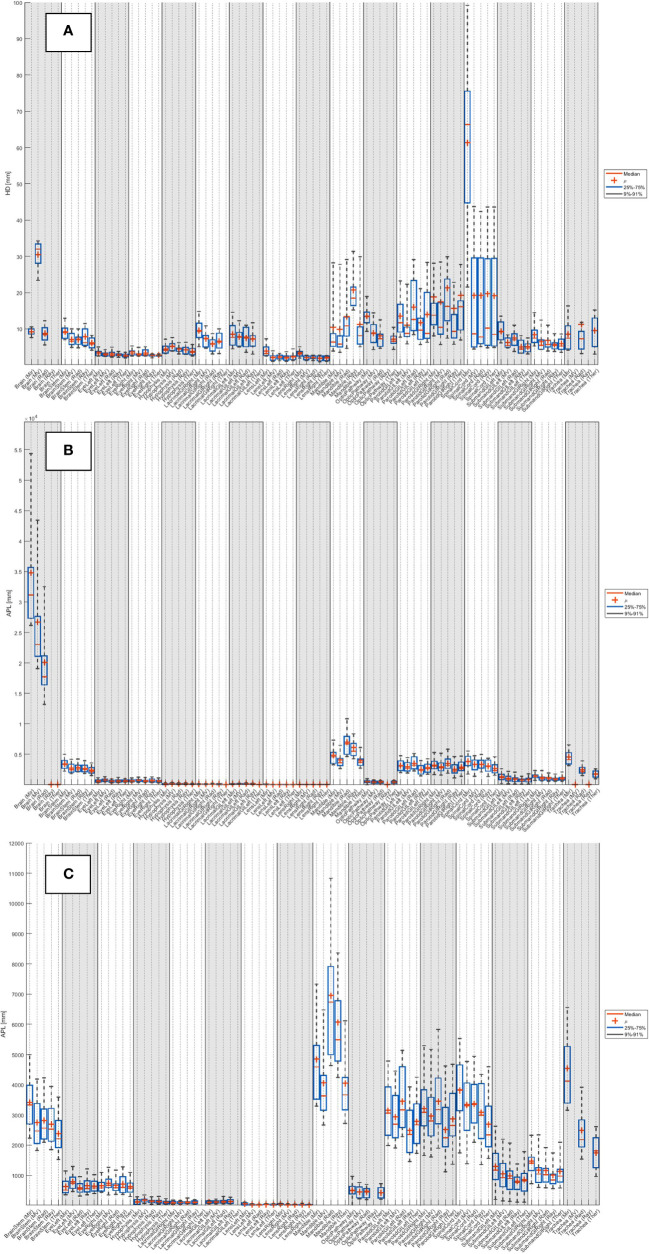
Distribution of **(A)** HD and **(B)** APL (with no added tolerance) across the 20 patients for each system, for the 19 organs at risk routinely contoured for head and neck. **(C)** Zoom of APL.

The median HDs and APLs for all systems across the 20 patients are detailed in **Supplementary Table 3** (breast), **Supplementary Table 5** (head and neck), **Supplementary Table 7** (lung) and **Supplementary Table 9** (prostate). The distribution of results across the 20 patients is shown as boxplots in **Supplementary Figure 3** (breast), **Figure 3** (head and neck), **Supplementary Figure 5** (lung) and **Supplementary Figure 7** (prostate).

### 3.2 Timing comparisons

For the expert contours, the average time (mean ± standard deviation) required to manually draw each anatomical site was found to be: breast 22 ± 4 mins; head and neck 97 ± 24 mins; lung 26 ± 6 mins; prostate 42 ± 11 mins. Examples of the contours produced by the AI contours, which require correction, is shown in **Figure 1**.

It can be seen from **Table 2** that, even after accounting for correction times, all systems offer time savings, ranging between: Breast 14.2-20.6 mins; head and neck 74.3-92.6 mins; lung 20.0-25.6 mins; prostate 34.6-41.9 mins.

**Table 2:** Mean time needed to correct AI-generated contours and time saving compared to manual contouring, for three patients and five different AI contouring solutions.

		Mean time					
		Manual Institution	Correction Mirada	Correction MVision	Correction Radformation	Correction RayStation	Correction Therapanacea
Breast	No. structures	10	8	8	10	5	10
	Time for 10 structures [min]	22	7.5	1.6	7.8	3.1	1.4
	Saving [min/%]		14.5/66.0%	20.4/92.8%	14.2/64.4%	18.9/86.0%	20.6/93.7%
Head and neck	No. structures	19	27	27	27	26	30
	Time for 19 structures [min]	97	8.2	9.8	22.7	4.6	4.4
	Saving [min/%]		88.8/91.6%	87.2/89.9%	74.3/76.6%	92.4/95.3%	92.6/95.4%
Lung	No. structures	6	6	6	6	5	6
	Time for 6 structures [min]	26	5.2	1.2	6.0	1.5	0.4
	Saving [min/%]		20.8/80.1%	24.9/95.6%	20.0/76.8%	24.5/94.4%	25.6/98.4%
Prostate	No. structures	10	8	9	9	5	10
	Time for 10 structures [min]	42	7.4	0.3	4.3	5.2	0.1
	Saving [min/%]		34.6/82.3%	41.7/99.3%	37.7/89.7%	36.8/87.6%	41.9/99.7%

The saving refers to the time saved computed by finding the mean time correct each contour, multiplied by the number of contours drawn by the manual.

## 4 Discussion

### 4.1 Overview

Auto-segmentation offers the opportunity to reduce inter- and intra-observer variability in contouring, as well as to reduce the time taken to conduct this manual task. It has been shown that AI offers an improvement in the quality of contours compared to atlas-based solutions, but to date there have been very few benchmarking tests to compare different commercial AI auto-segmentation solutions against a common dataset. The AAPM organized Grand Challenges at the 2017 (19) and 2019 (20) meetings on thoracic CT auto-segmentation and head and neck MRI segmentations, respectively, but tests on the systems currently available on the market is lacking. In this study we compared five commercial AI auto-segmentation solutions (Mirada, MVision, Radformation, RayStation and TheraPanacea) to generate organ at risk contours (a total of 45 structures) for 20 breast, 20 head and neck, 20 lung and 20 prostate patients. These AI contours were compared to contours drawn manually by a radiation oncologist.

### 4.2 Number of structures

There was a wide variation in the total number of structures that each AI contouring software can produce. Not all were assessed in this study because not all structures are routinely contoured in our clinic, so readers should inspect the details in **Table 1** for further information. Care should also be taken to review the guidelines that each system follows, so that they align with those of the clinic.

In this study only OARs were assessed, but when selecting an appropriate AI contouring solution for clinical use it may also be important to consider whether the system can contour lymph node levels, to aid with drawing of the CTV. Mir, MV, Ray and Ther can generate axillary lymph nodes, including levels 1-4, interpectoral and internal mammary nodes. MV, Rad, Ray and Ther can generate head and neck lymph nodes, including levels 1-7, with MV also contouring levels 9-10.

Another consideration for a clinic is that the AI contouring solutions generate more contours that are drawn in routine clinical use. While this may provide new insights, such as revealing doses to previously uncontoured organs that could be correlated with toxicity, they will also require additional editing and thus the time saved by the AI solution will decrease.

### 4.3 Timing comparisons

As can be seen in the results, all commercial auto-segmentation solutions offer significant time savings compared to manual contouring. Comparing the time to manually draw the contours to the time to perform corrections to the auto-segmentations, relative savings of 64-99% are possibly achieved depending on the anatomical site and commercial solution used. In general, minimal corrections are required for the structures involved in treating the breast, lung or prostate, while using any of the software for head and neck leads to an average saving of at least 74 mins. The time to correct any given structure ranged from 0 secs (lens) to 206 secs (bowels), the latter of which is comparable to the time to manually contour. As stated in the introduction, one of the primary motivations for introducing auto-segmentation is for time saving, and on this matter all systems offer an advantage over manual contouring. However, as shown by this range, sometimes it may be faster to manually contour the structure rather than editing an AI contour.

As stated in the Methods, if a system does not contour all the structures, it is assigned the average correction time from the other systems. This approach was utilized to allow a fair comparison between systems. For example, Ray does not contour liver or bowels, both of which required substantial corrections in our testing and thus led to longer correction times for the other systems.

### 4.4 Geometric comparison indices

Despite numerous studies showing that volumetric Dice similarity coefficient (vDSC) and Hausdorff distance (HD) do not correlate well with contour quality, they remain commonplace in the literature and thus the results in this study can be compared with previous works. They were therefore included alongside the more appropriate, but less well understood, surface Dice similarity coefficient (sDSC) and Added Path Length (APL) metrics. In this study there are too many structures to compare each one individually with literature, so general comments are made on anatomical sites as well as on outliers.

#### 4.4.1 Volumetric Dice similarity coefficient (vDSC) and surface Dice similarity coefficient (sDSC)

Van Dijk et al. (55) classified vDSC scores into good (vDSC>0.8), good-intermediate (0.7<vDSC<0.8), intermediate (0.6<vDSC<0.7), intermediate-poor (0.5<vDSC<0.6), poor (vDSC<0.5). From **Supplementary Tables 2**, **4**, **6** and **8**, averaged across all structures, the median vDSCs would be classified as good for all systems. However, it should be noted that vDSC scores are relative and highly dependent on the structure volume. A vDSC of 0.8 would be considered exceptionally good for small structures such as the optic nerves, while such a value would be considered very poor for large structures such as the brain or lungs. As such, comments on specific structures, for which there are extensive results in literature or for whom there were outliers, are made in the separate anatomical site sections below.

Due to its relatively recent introduction, there are few reported sDSC values in literature. Vaassen et al. (42) show sDSC values considerably less than vDSC, with the esophagus showing the most spread (0.07-0.94) and a median range of 0.66-0.97 for heart, lungs, mediastinum and spinal cord. From **Supplementary Tables 2**, **4**, **6** and **8**, averaged across all structures, the median sDSCs for all systems appear to be slightly lower than the results of Vaassen: Mir 0.47; MV 0.57; Rad 0.51; Ray 0.54; Ther 0.59; while the maximum results for each system are also not as high: Mir 0.70; MV 0.76; Rad 0.74; Ray 0.81; Ther 0.81.

#### 4.4.2 Hausdorff distance (HD) and Added Path Length (APL)

Van Dijk et al. (55) classified mean HDs into: good-intermediate HD<4mm, intermediate 4mm<HD<6mm, poor 6mm<HD<8mm and very poor (HD>8mm). From **Supplementary Tables 3**, **5**, **7** and **9**, averaged across all structures, the median maximum HDs in our study were found to be: Mir 13.4 mm; MV 10.7 mm; Rad 10.8 mm; Ray 12.0 mm; Ther 9.7 mm.

In computation of the APL, Vaassen et al. found that mediastinum had the highest value (826 cm [range 290–2441 cm], compared to a median range of 24–413 cm for heart, lungs, esophagus and spinal cord (42). In our study, from **Supplementary Tables 3**, **5**, **7** and **9**, averaged across all structures, the median APLs were all within this range: Mir 411.7 cm; MV 331.4 cm; Rad 342.5 cm; Ray 356.5 cm; Ther 355.9 cm.

### 4.5 Breast and lung cases

For the breast cases, there was a wide variation in the breast contour agreement, for all systems (across all systems, range 0.11-0.95). This is not unexpected because the limits of the breast are not clearly visible on CT and are difficult to define. Very high vDSC scores were observed for lungs (all systems ≥ 0.95) and liver (all systems ≥ 0.96). High values are also seen in literature for these structures (lungs 0.99, liver 0.98 (56)) whose boundaries are well defined and can be contoured by automated grey value thresholding. The esophagus demonstrates a variation between systems, which is evident in both the breast and lung cases. The clear outlier are the contours from Mir, but this is expected as the system contours much more superiorly than the expert.

### 4.6 Head and neck cases

The HD for brain was larger for MV than the other systems as they intentionally do not include the brainstem, which is included as part of the expert structure. The lacrimal gland vDSCs found for the different systems ranged between 0.52-0.61, which is slightly lower than what has been previously reported 0.69 (41). The lacrimal gland results have a wide spread, likely due to the difficulty in defining the contour boundaries. Lens vDSCs were found to be 0.56-0.83, with only Mir lying outside of previous reported values 0.67-0.99 (56–58). The mandible contour from Rad showed the largest APL as it often contoured parts of the teeth. Poor vDSC scores have been previously reported in literature for the optic chiasm [e.g. vDSC 0.37-0.63 (56, 59, 60)], likely due to the difficulty of its visualization on CT. By assessing the optic pathway, rather than individually the chiasm and optic nerves, the systems in this study achieved vDSCs between 0.53-0.68, with Ray excluded because it does not contour the chiasm. Segmentation of the parotid in CT images is challenging due to the irregular shape, poorly visible boundaries (59), and (possibly) dental artifacts. Parotid vDSCs were found to range between 0.81-0.88 for this study, which is in the upper range as reported in literature 0.57-0.95 (56, 61–72). The range of submandibular glands vDSCs (0.75-0.90) produced by the AI systems were all towards the upper end of previously reported values 0.60-0.88 (56, 57, 73, 74). The spinal cord vDSCs in this study ranged between 0.69-0.83, which fits into the range reported in literature previously 0.62-0.90 (57, 61, 62, 70, 75; La 58, 76, 77). Spinal cord HDs were much higher with Mir than the other systems because it did not contour along the whole length of the CT scan.

### 4.7 Prostate cases

Typically vDSC scores in the pelvic region have ranged between 0.60-0.99 (45, 54, 56, 78, 79), and similar values were found in our prostate cases: Mir 0.54-0.95; MV 0.68-0.97; Rad 0.66-0.97; Ray 0.85-0.95; Ther 0.52-0.97. The lowest values were for the penile root (vDSC 0.54-0.71) which is difficult to visualize on CT, and the bowels (vDSC 0.59-0.76) and sigmoid (vDSC 0.52-0.77), which are generally contoured more precisely by the AI solutions. It should be noted that none of these three structures are contoured by Ray, so the lower bound of its range above is higher.

### 4.8 Usefulness of metrics and study limitations

As detailed in the introduction, as well as standardization of practice one of the primary motivations for the introduction of an auto-segmentation solution is to save time. In contouring, time-saving depends on three main aspects; the visibility of the OAR boundaries; the volume of the OAR; and the delineation tools used for manual contouring (42). For example, the esophagus has poorly visible boundaries, which is reflected by poor vDSC (**Supplementary Figure 2**). Additionally, the esophagus is volumetrically a relatively small organ and vDSC is normalized by volume, leading to further suppression of values. However, because it is a small structure the number of slices it covers is few and the absolute time needed to adjust the contour is low. It has been shown that APL is a useful metric for such assessments because it takes all of the above issues into account, which is shown by the low values for esophagus in **Supplementary Figure 3**.

A limitation of the study is that the gold standard, to which all systems are being compared, is a single set of contours drawn by a single radiation oncologist. Despite being drawn by experienced radiation oncologists following strict guidelines, there will inevitably be different interpretations of the guidelines and inter-observer variations (which can be substantial). A more complete study would be to form the expert contours from the average of multiple independently drawn structures from different radiation oncologists, but the additional time this requires meant it was not feasible to do this while simultaneously assessing the most contemporary models for each system.

Another limitation of the work presented is the contemporary nature of the field. Since the models were run on the datasets, all systems have updated their models and offer additional structures and improvements to the contouring quality (for instance, Mir released a head and neck lymph node model after the analysis was performed). Users should bear in mind that the data presented in this study therefore reflects the status of each solution in April 2022.

In this work it was assumed that the time to generate the contours is zero for all systems because the operation can be performed in the background. However, this is not the case for Ray, in which the user must import the CT dataset into the TPS, select the appropriate deep learning algorithm and run it to generate the contours. Additionally, in some systems, such as Rad, the user is prevented from exporting the RT structure set from the AI auto-segmentation software unless a manual review of all AI-generated contours has been performed across all slices. In this work these times were not accounted for.

It has been discussed that clinical acceptance of an AI auto-segmentation solution requires evaluation in multiple domains (39, 80). In addition to the quantitative geometric comparison and timing efficiencies assessed in this work, it is desirable to conduct a qualitative evaluation by the end user, as well as assess the clinical impact in terms of OAR and target doses. However, the intention of this work is to provide guidance for centers wishing to purchase a commercial AI segmentation solution and, as such, it is critical for the information to be contemporary and relevant. As stated in Section 2.2, all commercial products undergo regular updates so it was not feasible to perform assessments in all domains in a timely manner. These are planned as future work.

### 4.9 Selection of an AI auto-segmentation solution

As well as the number of structures (which, it should be noted, is often customizable between anatomical sites for most systems), geometric indices and timing savings detailed above, there are other parameters to consider when selecting an appropriate AI auto-segmentation solution. The cost should be considered, which is typically either a fixed annual fee or on a per-patient basis. If utilizing the per-patient model, users should bear in mind that the number of times the model may be utilized may be higher than the number of patients if replans are common or if the auto-segmentation is run on daily CBCT images. Additionally, the user should ensure that the contours offered, and the guidelines they follow, are aligned with the structures that are required by their clinical practice.

Another important consideration is the connectivity of the solution and how it integrates within existing clinical workflows. Excessive exporting and importing into current contouring software and TPSs increases the potential for data corruption and impacts on the potential time-saving. Also, while it is critical that all AI-generated contours are reviewed, the user may prefer to perform this to do this in their own contouring software, which is not possible with all systems.

## 5 Conclusions

It can be concluded that all five commercial AI auto-segmentation solutions assessed in this work, Mirada, MVision, Radformation, RayStation and TheraPanacea, offer significant time-saving compared to manual contouring, while the quality of structures generated is very good and in line with previous literature. While each system performs at different levels, the introduction of any commercial AI auto-contouring solution offers the potential for more contours to be drawn, improved consistency and standardization, minimization of inter-observer variability and time saving. In the future, a separate evaluation of AI contouring of lymph nodes as well as an inter-observer validation of the contours will be performed.

## Data availability statement

The original contributions presented in the study are included in the article/**Supplementary Material**. Further inquiries can be directed to the corresponding author.

## Ethics statement

The studies involving human participants were reviewed and approved by Department of Radiation Oncology, German Oncology Center. The patients/participants provided their written informed consent to participate in this study.

## Author contributions

Study design was curated by PD, CZ and EK. Processing of patients was conducted by PD, YR, SC, AL, MP and MB. Contouring and corrections was conducted by KF, IS and EK. Data was analyzed by PD. Manuscript written by PD. All authors contributed to the article and approved the submitted version.
