# Supplementary material for: A clinical evaluation of the performance of five commercial artificial intelligence contouring systems for radiotherapy
Source: Front Oncol. 2023 Aug 4;13:1213068. doi: 10.3389/fonc.2023.1213068 (PMC10436522; doi:10.3389/fonc.2023.1213068)
Supplement: Supplementary file 1 [file Table_1.docx]

Supplementary Material

A clinical evaluation of the performance of five commercial artificial intelligence contouring systems for radiotherapy

Paul J Doolan^1*^, Stefanie Charalambous^2^, Yiannis Roussakis^1^, Agnes Leczynski^2^, Mary Peratikou^2^, Melka Benjamin^2^, Konstantinos Ferentinos^2,3^, Iosif Strouthos^2,3^, Constantinos Zamboglou^2,3,4^, Efstratios Karagiannis^2,3^

*** Correspondence:** Corresponding Author: [paul.doolan@goc.com.cy](mailto:paul.doolan@goc.com.cy)

# Supplementary figure captions

Supplementary Fig. 1. A demonstration of the different contouring approaches to the optic nerves and optic chiasm. (a) Expert contours alone; (b) expert and one system; (c) expert and another system. In this work a structure called ‘optic pathway’ was assessed, which combined the three individual structures: optic nerves and optic chiasm.

Supplementary Fig. 2. Distribution of (a) vDSC and (b) sDSC (with no added tolerance) across the 20 patients for each system, for the 10 organs at risk routinely contoured for breasts.

Supplementary Fig. 3. Distribution of (a) HD and (b) APL (with no added tolerance) across the 20 patients for each system, for the 10 organs at risk routinely contoured for breasts.

Supplementary Fig. 4. Distribution of (a) vDSC and (b) sDSC (with no added tolerance) across the 20 patients for each system, for the six organs at risk routinely contoured for lungs.

Supplementary Fig. 5. Distribution of (a) HD and (b) APL (with no added tolerance) across the 20 patients for each system, for the six organs at risk routinely contoured for lung.

Supplementary Fig. 6. Distribution of (a) vDSC and (b) sDSC (with no added tolerance) across the 20 patients for each system, for the 10 organs at risk routinely contoured for prostates.

Supplementary Fig. 7. Distribution of (a) HD and (b) APL (with no added tolerance) across the 20 patients for each system, for the 10 organs at risk routinely contoured for prostates. (c) Zoom of APL.

# Supplementary tables

Supplementary Table 1. Total list of OAR structures included in the CT models for each commercial solution (as of April 2022).

| Institution structures | AI contouring software structures | | | | | | |
| --- | --- | --- | --- | --- | --- | --- | --- |
|  | Mirada | MVision | | Radformation | | RayStation | Therapanacea |
| BodySurface | External | Body | | External | | External | External_Contour |
|  |  |  | |  | |  | Anal_Canal |
|  | A_Aorta | A_Aorta | | A_Aorta | |  | A_Aorta_Thorax |
|  |  |  | | A_Aorta_Asc | |  |  |
|  |  |  | | A_Aorta_Dsc | |  |  |
|  | AdrenalGlnd_L |  | |  | |  |  |
|  | AdrenalGlnd_R |  | |  | |  |  |
|  |  | A_LAD | | A_LAD | | A_LAD |  |
|  | Carotid_L | A_Carotid_L | |  | |  |  |
|  | Carotid_R | A_Carotid_R | |  | |  |  |
|  | Arytenoid_L | Arytenoid_L | |  | |  |  |
|  | Arytenoid_R | Arytenoid_R | |  | |  |  |
| Bladder | Bladder | Bladder | | Bladder | | Bladder | Bladder |
|  | Bone_Ilium_L |  | | Bone_Ilium_L | |  | Bone_Ilium_L |
|  | Bone_Ilium_R |  | | Bone_Ilium_R | |  | Bone_Ilium_R |
|  | Bone_Ischium_L |  | |  | |  |  |
|  | Bone_Ischium_R |  | |  | |  |  |
|  | Bone_Lumbar |  | |  | |  |  |
|  | Bone_Pelvic | Bone_Pelvic | |  | |  |  |
|  | Bone_Pubic_L |  | |  | |  |  |
|  | Bone_Pubic_R |  | |  | |  |  |
| Bowels | Bag_Bowel | Bag_Bowel | | Bowel_Bag | |  | Bag_Bowel |
|  | Total_Bowel | Bowel_Large | |  | |  |  |
|  |  | Bowel_Small | |  | |  |  |
|  | BrachP_L | BrachialPlex_L | | BrachialPlex_L | |  | BrachialPlex_L |
|  | BrachP_R | BrachialPlex_R | | BrachialPlex_R | |  | BrachialPlex_R |
| Brain | Brain | Brain | | Brain | |  | Encephalon |
| BrainStem | Brainstem | Brainstem | | Brainstem | | Brainstem | Brainstem |
| Breast_L | Breast_L | Breast_L | | Breast_L | | Breast_L | Breast_L |
| Breast_R | Breast_R | Breast_R | | Breast_R | | Breast_R | Breast_R |
|  |  | Bronchus_Prox | |  | |  |  |
|  |  |  | | Bronchus | |  |  |
|  |  |  | |  | |  | Bronchus_L |
|  |  |  | |  | |  | Bronchus_R |
|  | Buccalmucosa_L | Buccal_Mucosa_L | |  | |  |  |
|  | Buccalmucosa_R | Buccal_Mucosa_R | |  | |  |  |
|  |  |  | | Carina | |  |  |
| CaudaEquina |  |  | | CaudaEquina | |  |  |
|  | Cavity_Oral | Cavity_Oral | | Cavity_Oral | | Cavity_Oral | Cavity_Oral |
|  | Cerebellum |  | |  | |  | Cerebellum |
|  | Cerebrum |  | |  | |  |  |
|  |  | Chestwall_L | | Chestwall_OAR | |  |  |
|  |  | Chestwall_R | |  |  |  |  |
|  | Cochlea_L | Cochlea_L | | Cochlea_L | | Cochlea_L | Cochlea_L |
|  | Cochlea_R | Cochlea_R | | Cochlea_R | | Cochlea_R | Cochlea_R |
|  | Inlet_Cricophar | Cricophar_inlet | |  | |  |  |
| Chiasma | Optic_Chiasm | OpticChiasm | | OpticChiasm | |  | OpticChiasm |
|  |  | OpticChiasm_cnv | |  | |  |  |
|  | Duodenum |  | |  | |  |  |
|  |  | Eye_Ant_L | |  | |  |  |
|  |  | Eye_Ant_R | |  | |  |  |
| EyeLeft | Orbit_L | Eye_L | | Eye_L | | Eye_L | Eye_L |
|  |  | Eye_Post_L | |  | |  |  |
|  |  | Eye_Post_R | |  | |  |  |
| EyeRight | Orbit_R | Eye_R | | Eye_R | | Eye_R | Eye_R |
| FemoralHead_L | Femur_Head_L | Femur_L | | Femur_L | | Femur_Head_L | Femur_Head_L |
| FemoralHead_R | Femur_Head_R | Femur_R | | Femur_R | | Femur_Head_R | Femur_Head_R |
|  | FemurHeadNeck_L |  | | Femur_RTOG_L | |  |  |
|  | FemurHeadNeck_R |  | | Femur_RTOG_R | |  |  |
|  |  | |  | |  | Fossa_Posterior |  |
|  | Gallbladder | |  | |  |  |  |
|  | GlotticArea | | Glottis | |  | Glottis |  |
| Heart | Heart | | Heart | | Heart | Heart | Heart |
|  | Heart_Thorax | | Heart+A_Pulm | |  |  |  |
| Humerus_L |  | | Humerus_L | | Humerus_L | HumeralHead_L | Humerus_Head_L |
| Humerus_R |  | | Humerus_R | | Humerus_R | HumeralHead_R | Humerus_Head_R |
| Hyoid |  | |  | |  |  |  |
| Hypophysis | Pituitary | | Pituitary | | Pituitary | Pituitary | Hypophysis |
| InnerEarLeft | IAM_L | |  | | Ear_Internal_L |  |  |
| InnerEarRight | IAM_R | |  | | Ear_Internal_R |  |  |
|  | Kidney_L | | Kidney_L | |  | Kidney_L | Kidney_L |
|  | Kidney_R | | Kidney_R | |  | Kidney_R | Kidney_R |
| LacrimalGILeft | Lacrimal_L | | Glnd_Lacrimal_L | | Glnd_Lacrimal_L | Glnd_Lacrimal_L |  |
| LacrimalGIRight | Lacrimal_R | | Glnd_Lacrimal_R | | Glnd_Lacrimal_R | Glnd_Lacrimal_R |  |
|  | Larynx | |  | | Larynx |  | Larynx |
|  | Supraglottic Larynx | |  | |  | Larynx_SG |  |
|  |  | | LN_Axillary_L | |  |  |  |
|  |  | | LN_Axillary_R | |  |  |  |
|  | LN_Ax_L1_L | | LN_Breast_L1_L | |  | LN_Ax_L1_L | LN_Ax_L1_L |
|  | LN_Ax_L1_R | | LN_Breast_L1_R | |  | LN_Ax_L1_R | LN_Ax_L1_R |
|  | LN_Ax_L2_L | | LN_Breast_L2_L | |  | LN_Ax_L2_L | LN_Ax_L2_L |
|  | LN_Ax_L2_R | | LN_Breast_L2_R | |  | LN_Ax_L2_R | LN_Ax_L2_R |
|  | LN_Ax_L3_L | | LN_Breast_L3_L | |  | LN_Ax_L3_L | LN_Ax_L3_L |
|  | LN_Ax_L3_R | | LN_Breast_L3_R | |  | LN_Ax_L3_R | LN_Ax_L3_R |
|  | LN_Ax_L4_L | | LN_Breast_L4_L | |  | LN_Ax_L4_L |  |
|  | LN_Ax_L4_R | | LN_Breast_L4_R | |  | LN_Ax_L4_R |  |
|  | LN_Interpect_L | | LN_Intpect_L | |  | LN_Ax_Pectoral_L | LN_Interpectoral_L |
|  | LN_Interpect_R | | LN_Intpect_R | |  | LN_Ax_Pectoral_R | LN_Interpectoral_R |
|  |  | | LN_IMN_IC4_L | |  |  |  |
|  |  | | LN_IMN_IC4_R | |  |  |  |
|  | IMC_L | | LN_IMN_L | |  | LN_IMN_L | LN_IMN_L |
|  | IMC_R | | LN_IMN_R | |  | LN_IMN_R | LN_IMN_R |
|  |  | |  | |  |  | LN_Sclav_L |
|  |  | |  | |  |  | LN_Sclav_R |
| Level1A |  | | LN_Neck_IA | | LN_Neck_IA |  | LN_Neck_IA |
| Level1BLeft |  | | LN_Neck_IB_L | | LN_Neck_IB-V_L |  | LN_Neck_IB_L |
| Level1BRight |  | | LN_Neck_IB_R | | LN_Neck_IB-V_R |  | LN_Neck_IB_R |
| Level2Left |  | | LN_Neck_II_L | | LN_Neck_II_L |  | LN_Neck_II_L |
| Level2Right |  | | LN_Neck_II_R | | LN_Neck_II_R |  | LN_Neck_II_R |
| Level3Left |  | | LN_Neck_III_L | | LN_Neck_III_L |  | LN_Neck_III_L |
| Level3Right |  | | LN_Neck_III_R | | LN_Neck_III_R |  | LN_Neck_III_R |
| Level4Left |  | | LN_Neck_IVA_L | | LN_Neck_IV_L |  | LN_Neck_IVA_L |
| Level4Right |  | | LN_Neck_IVA_R | | LN_Neck_IV_R |  | LN_Neck_IVA_R |
|  |  | | LN_Neck_IVB_L | | LN_Neck_II-IV_L |  | LN_Neck_IVB_L |
|  |  | | LN_Neck_IVB_R | | LN_Neck_II-IV_R |  | LN_Neck_IVB_R |
| Level5Left |  | | LN_Neck_V_L | |  |  | LN_Neck_V_L |
| Level5Right |  | | LN_Neck_V_R | |  |  | LN_Neck_V_R |
|  |  | | LN_Neck_VC_L | |  |  |  |
|  |  | | LN_Neck_VC_R | |  |  |  |
|  |  | | LN_Neck_VIA | | LN_Neck_VIA |  |  |
|  |  | | LN_Neck_VIB | |  |  |  |
|  |  | | LN_Neck_VIIA_L | | LN_Neck_VIIA_L |  | LN_Neck_VIIA_L |
|  |  | | LN_Neck_VIIA_R | | LN_Neck_VIIA_R |  | LN_Neck_VIIA_R |
|  |  | | LN_Neck_VIIB_L | | LN_Neck_VIIB_L |  | LN_Neck_VIIB_L |
|  |  | | LN_Neck_VIIB_R | | LN_Neck_VIIB_R |  | LN_Neck_VIIB_R |
|  |  | | LN_Neck_IX_L | |  |  |  |
|  |  | | LN_Neck_IX_R | |  |  |  |
|  |  | | LN_Neck_XA_L | |  |  |  |
|  |  | | LN_Neck_XA_R | |  |  |  |
|  |  | | LN_Neck_XB_L | |  |  |  |
|  |  | | LN_Neck_XB_R | |  |  |  |
|  |  | | LN_Pivotal | |  |  |  |
|  | LN_Pelvic | |  | |  |  |  |
|  |  | | LN_RTOG | |  |  |  |
| LensLeft | Lens_L | | Lens_L | | Lens_L | Lens_L | Lens_L |
| LensRight | Lens_R | | Lens_R | | Lens_R | Lens_R | Lens_R |
|  | Lips | | Lips | | Lips |  | Lips |
| Liver | Liver | | Liver | | Liver | Liver | Liver |
| Lung_L | Lung_L | | Lung_L | | Lung_L | Lung_L | Lung_L |
| Lung_R | Lung_R | | Lung_R | | Lung_R | Lung_R | Lung_R |
| Mandible | Mandible | | Bone_Mandible | | Bone_Mandible | Bone_Mandible | Bone_Mandible |
| MandibleLeft |  | |  | |  |  |  |
| MandibleRight |  | |  | |  |  |  |
|  |  | |  | | Marrow_Ilium_L |  |  |
|  |  | |  | | Marrow_Ilium_R |  |  |
|  |  | |  | |  |  | MedullaryCanal |
|  |  | | Musc_Coccygeus_L | |  |  |  |
|  |  | | Musc_Coccygeus_R | |  |  |  |
|  | Musc_Constrict | | Musc_Constrict | | Musc_Constrict |  |  |
|  |  | | Musc_Iliacus_L | |  |  |  |
|  |  | | Musc_Iliacus_R | |  |  |  |
|  |  | | Musc_Obt_Int_L | |  |  |  |
|  |  | | Musc_Obt_Int_R | |  |  |  |
|  |  | | Musc_Pirifor_L | |  |  |  |
|  |  | | Musc_Pirifor_R | |  |  |  |
|  |  | | Musc_Psoas_Maj_L | |  |  |  |
|  |  | | Musc_Psoas_Maj_R | |  |  |  |
|  |  | |  | |  | Nasolacrimal_Duct_L |  |
|  |  | |  | |  | Nasolacrimal_Duct_R |  |
|  |  | |  | |  | Nasopharynx |  |
| Oesophagus | Oesophagus | | Esophagus | | Esophagus | Esophagus | Esophagus |
|  | Esophagus_S | | Esophagus_S | | Esophagus | Esophagus_S | Esophagus |
| OpticNerveLeft | Opt_N_L | | OpticNrv_L | | OpticNrv_L | OpticNrv_L | OpticNrv_L |
| OpticNerveRight | Opt_N_R | | OpticNrv_R | | OpticNrv_R | OpticNrv_R | OpticNrv_R |
|  |  | | OpticNrv_cnv_L | |  |  |  |
|  |  | | OpticNrv_cnv_R | |  |  |  |
|  |  | |  | |  | Oropharynx |  |
|  | Pancreas | | Pancreas | |  | Pancreas |  |
| ParotidGILeft | Parotid_L | | Parotid_L | | Parotid_L | Parotid_L | Parotid_L |
| ParotidGIRight | Parotid_R | | Parotid_R | | Parotid_R | Parotid_R | Parotid_R |
|  | PelvicRim | |  | |  |  |  |
| PenileRoot | PenileBulb | | PenileBulb | | PenileBulb |  | PenileBulb |
|  |  | |  | |  |  | CTVN_Prostate |
| Prostate | Prostate | | Prostate | | Prostate | Prostate | Prostate |
| Rectum | Rectum | | Rectum | | Rectum | Anorectum | Rectum |
|  | Sacrococcyx | |  | |  |  |  |
|  |  | | Sacrum | |  |  |  |
| SV | SeminalVes | | SeminalVes | | SeminalVes |  | SeminalVes |
| Sigmoid |  | | RectoSigmoid | |  |  | Colon_Sigmoid |
| SpinalCanal | SpinalCanal | | SpinalCanal | | SpinalCanal | SpinalCanal |  |
| SpinalCord | Spinal_Cord | | SpinalCord | | SpinalCord | SpinalCord | SpinalCord |
|  | Spleen | | Spleen | | Spleen | Spleen |  |
| SternocleidoLeft |  | |  | |  |  |  |
| SternocleidoRight |  | |  | |  |  |  |
|  |  | |  | | Bone_Sternum | Sternum |  |
|  |  | | Stomach | | Stomach | Stomach |  |
| SubmandGILeft | Submandibular_L | | Glnd_Submand_L | | Glnd_Submand_L | Glnd_Submand_L | Glnd_Submand_L |
| SubmandGIRight | Submandibular_R | | Glnd_Submand_R | | Glnd_Submand_R | Glnd_Submand_R | Glnd_Submand_R |
|  |  | |  | |  | Joint_TM_L | Joint_TM_L |
|  |  | |  | |  | Joint_TM_R | Joint_TM_R |
|  |  | |  | |  | Tongue_Base |  |
|  | Thyroid | | Glnd_Thyroid | | Glnd_Thyroid | ThyroidGland | Glnd_Thyroid |
| Trachea | Trachea | | Trachea | | Trachea | Trachea | Trachea |
|  |  | | Trachea_Prox | |  |  |  |
|  | V_Venacava_I | | V_Venacava_I | |  |  |  |
|  |  | | V_Venacava_S | | V_VenaCava_S |  |  |
|  | VB_L4 | L4_VB | |  | |  |  |
|  | VB_L5 | L5_VB | |  | |  |  |
|  | Iliac_Vessel | |  | |  |  |  |
|  |  | | Vessels_L | |  |  |  |
|  |  | | Vessels_Long_L | |  |  |  |
|  |  | | Vessels_Long_R | |  |  |  |
|  |  | | Vessels_R | |  |  |  |
| Totals | | | | | | | |
| Institution  58 | Mirada  99 | | MVision  142 | | Radformation  83 | RayStation  67 | Therapanacea  86 |

Supplementary Table 2. Median volumetric Dice (vDSC) and median surface Dice (sDSC, with no additional tolerance) similarity coefficients for twenty patients and five different AI contouring solutions, for breast models.

| Structure | System | | | | | | | | | |
| --- | --- | --- | --- | --- | --- | --- | --- | --- | --- | --- |
|  | Mirada | | MVision | | Radformation | | RayStation | | Therapanacea | |
|  | vDSC | sDSC | vDSC | sDSC | vDSC | sDSC | vDSC | sDSC | vDSC | sDSC |
| Breast_L | 0.857 | 0.254 | 0.895 | 0.350 | 0.803 | 0.160 | 0.832 | 0.137 | 0.905 | 0.321 |
| Breast_R | 0.866 | 0.246 | 0.907 | 0.320 | 0.835 | 0.164 | 0.841 | 0.152 | 0.877 | 0.290 |
| Heart | 0.937 | 0.422 | 0.954 | 0.491 | 0.951 | 0.455 | 0.948 | 0.455 | 0.941 | 0.478 |
| Humerus^a^ | - | - | 0.907 | 0.660 | 0.910 | 0.682 | 0.813 | 0.533 | 0.862 | 0.632 |
| Liver | 0.961 | 0.554 | 0.965 | 0.595 | 0.959 | 0.543 | 0.964 | 0.581 | 0.967 | 0.590 |
| Lung_L | 0.966 | 0.561 | 0.968 | 0.607 | 0.960 | 0.536 | 0.960 | 0.546 | 0.969 | 0.612 |
| Lung_R | 0.959 | 0.573 | 0.962 | 0.601 | 0.950 | 0.475 | 0.959 | 0.566 | 0.963 | 0.598 |
| Oesophagus | 0.734 | 0.499 | 0.793 | 0.588 | 0.755 | 0.510 | 0.809 | 0.637 | 0.840 | 0.629 |
| SpinalCanal | 0.822 | 0.516 | 0.83 | 0.481 | 0.842 | 0.534 | 0.838 | 0.531 | 0.846 | 0.548 |

^a^Only the ipsilateral Humerus is contoured clinically, so no laterality listed here. The indices listed are the means of left and right humerus median values.

Supplementary Table 3. Median Hausdorff distance (HD) (in mm) and median Added Path Length (APL, with no additional tolerance) coefficients for twenty patients and five different AI contouring solutions, for breast models.

| Structure | System | | | | | | | | | |
| --- | --- | --- | --- | --- | --- | --- | --- | --- | --- | --- |
|  | Mirada | | MVision | | Radformation | | RayStation | | Therapanacea | |
|  | HD | APL | HD | APL | HD | APL | HD | APL | HD | APL |
| Breast_L | 28.1 | 30380 | 22.8 | 25607 | 38.8 | 35340 | 28.0 | 36053 | 20.1 | 28181 |
| Breast_R | 30.2 | 27546 | 19.9 | 26008 | 36.1 | 33432 | 21.6 | 33918 | 29.7 | 28319 |
| Heart | 16.7 | 19236 | 10.8 | 17922 | 10.7 | 20252 | 12.0 | 19262 | 10.5 | 18115 |
| Humerus^a^ | - | - | 19.9 | 2670 | 20.6 | 2216 | 44.4 | 4520 | 36.9 | 3567 |
| Liver | 17.4 | 29255 | 18.4 | 25995 | 24.9 | 31248 | 22.7 | 26532 | 22.1 | 28043 |
| Lung_L | 24.1 | 35206 | 25.3 | 32763 | 24.6 | 37397 | 28.4 | 37980 | 26.8 | 32844 |
| Lung_R | 19.7 | 29982 | 20.4 | 27896 | 21.7 | 35266 | 23.4 | 31733 | 19.5 | 28851 |
| Oesophagus | 21.2 | 3522 | 19.1 | 2504 | 19.4 | 3200 | 13.6 | 2260 | 9.9 | 2441 |
| SpinalCanal | 9.2 | 5239 | 6.5 | 5293.5 | 6.3 | 5092 | 7.7 | 4959 | 6.7 | 5175 |

^a^Only the ipsilateral Humerus is contoured clinically, so no laterality listed here. The indices listed are the means of left and right humerus median values.

Supplementary Table 4. Median volumetric Dice (vDSC) and median surface Dice (sDSC, with no additional tolerance) similarity coefficients for twenty patients and five different AI contouring solutions, for head and neck models.

| Structure | System | | | | | | | | | | | | |
| --- | --- | --- | --- | --- | --- | --- | --- | --- | --- | --- | --- | --- | --- |
|  | Mirada | | MVision | | | Radformation | | RayStation | | | Therapanacea | | |
|  | vDSC | sDSC | vDSC | | sDSC | vDSC | sDSC | vDSC | | sDSC | vDSC | | sDSC |
| Brain | 0.970 | 0.493 | 0.965 | 0.609 | | 0.984 | 0.727 | - | - | | - | - | |
| BrainStem | 0.786 | 0.305 | 0.871 | 0.451 | | 0.861 | 0.396 | 0.865 | 0.462 | | 0.889 | 0.479 | |
| EyeLeft | 0.919 | 0.698 | 0.891 | 0.582 | | 0.920 | 0.700 | 0.918 | 0.68 | | 0.923 | 0.671 | |
| EyeRight | 0.917 | 0.658 | 0.897 | 0.600 | | 0.915 | 0.664 | 0.915 | 0.651 | | 0.925 | 0.687 | |
| Hypophysis | 0.555 | 0.421 | 0.359 | 0.236 | | 0.522 | 0.442 | 0.584 | 0.464 | | 0.681 | 0.551 | |
| LacrimalGILeft | 0.519 | 0.430 | 0.545 | 0.489 | | 0.545 | 0.447 | 0.548 | 0.508 | | - | - | |
| LacrimalGIRight | 0.605 | 0.534 | 0.556 | 0.502 | | 0.529 | 0.450 | 0.520 | 0.468 | | - | - | |
| LensLeft | 0.561 | 0.473 | 0.795 | 0.747 | | 0.781 | 0.736 | 0.831 | 0.771 | | 0.820 | 0.759 | |
| LensRight | 0.636 | 0.574 | 0.799 | 0.762 | | 0.783 | 0.710 | 0.823 | 0.807 | | 0.830 | 0.805 | |
| Mandible | 0.912 | 0.669 | 0.926 | 0.755 | | 0.856 | 0.513 | 0.878 | 0.619 | | 0.926 | 0.752 | |
| OpticPathway | 0.534 | 0.487 | 0.676 | 0.666 | | 0.591 | 0.546 | - | - | | 0.680 | 0.659 | |
| ParotidGILeft | 0.822 | 0.417 | 0.865 | 0.490 | | 0.831 | 0.419 | 0.877 | 0.539 | | 0.876 | 0.55 | |
| ParotidGIRight | 0.818 | 0.436 | 0.859 | 0.482 | | 0.834 | 0.397 | 0.880 | 0.578 | | 0.858 | 0.521 | |
| SpinalCord | 0.687 | 0.429 | 0.768 | 0.512 | | 0.764 | 0.446 | 0.794 | 0.513 | | 0.834 | 0.58 | |
| SubmandGILeft | 0.750 | 0.390 | 0.834 | 0.522 | | 0.854 | 0.584 | 0.888 | 0.635 | | 0.893 | 0.634 | |
| SubmandGIRight | 0.759 | 0.397 | 0.847 | 0.555 | | 0.861 | 0.583 | 0.871 | 0.614 | | 0.857 | 0.55 | |
| Trachea | 0.739 | 0.092 | - | - | | 0.857 | 0.476 | - | - | | 0.888 | 0.659 | |

Supplementary Table 5. Median Hausdorff distance (HD) and median Added Path Length (APL, with no additional tolerance) coefficients for twenty patients and five different AI contouring solutions, for head and neck models.

| Structure | System | | | | | | | | | | | | |
| --- | --- | --- | --- | --- | --- | --- | --- | --- | --- | --- | --- | --- | --- |
|  | Mirada | | MVision | | | Radformation | | RayStation | | | Therapanacea | | |
|  | HD | APL | HD | | APL | HD | APL | HD | | APL | HD | | APL |
| Brain | 9.3 | 31156 | 32.0 | 23021 | | 8.4 | 17726 | - | - | | - | - | |
| BrainStem | 8.9 | 3334 | 6.6 | 2468 | | 7.2 | 2563 | 6.2 | 2525 | | 5.8 | 2210 | |
| EyeLeft | 3.2 | 512 | 2.9 | 733 | | 2.7 | 543 | 2.7 | 559 | | 2.5 | 580 | |
| EyeRight | 3.2 | 581 | 2.9 | 666 | | 2.9 | 589 | 2.7 | 597 | | 2.7 | 571 | |
| Hypophysis | 4.1 | 110 | 5.1 | 177 | | 4.6 | 143 | 4.3 | 128 | | 3.5 | 97 | |
| LacrimalGILeft | 9.3 | 78 | 7.4 | 93 | | 6.0 | 96 | 6.4 | 102 | | - | - | |
| LacrimalGIRight | 7.7 | 91 | 7.7 | 105 | | 7.7 | 115 | 7.1 | 124 | | - | - | |
| LensLeft | 3.2 | 55 | 2.1 | 22 | | 2.2 | 18 | 2.0 | 23 | | 2.3 | 25 | |
| LensRight | 3.1 | 41 | 2.1 | 20 | | 2.1 | 20 | 2.0 | 24 | | 2.2 | 19 | |
| Mandible | 6.4 | 4586 | 5.8 | 3626 | | 10.8 | 6732 | 18.5 | 5490 | | 8.1 | 3665 | |
| OpticPathway | 13.4 | 493 | 8.7 | 456 | | 7.3 | 485 | - | - | | 6.6 | 419 | |
| ParotidGILeft | 11.7 | 3054 | 8.8 | 2693 | | 12.6 | 3169 | 9.7 | 2352 | | 8.8 | 2670 | |
| ParotidGIRight | 13.8 | 3084 | 10.5 | 2799 | | 16.2 | 3176 | 9.3 | 2243 | | 16.1 | 2654 | |
| SpinalCord | 66.4 | 3829 | 8.7 | 3307 | | 7.8 | 3338 | 10.2 | 2989 | | 8.5 | 2341 | |
| SubmandGILeft | 9.2 | 1173 | 5.4 | 919 | | 7.0 | 893 | 4.4 | 763 | | 4.7 | 818 | |
| SubmandGIRight | 7.5 | 1404 | 5.5 | 1044 | | 5.7 | 1024 | 5.5 | 845 | | 5.3 | 975 | |
| Trachea | 7.5 | 4117 | - | - | | 7.3 | 2185 | - | - | | 9.5 | 1814 | |

Supplementary Table 6. Median volumetric Dice (vDSC) and median surface Dice (sDSC, with no additional tolerance) similarity coefficients for twenty patients and five different AI contouring solutions, for lung models.

| Structure | System | | | | | | | | | | | | |
| --- | --- | --- | --- | --- | --- | --- | --- | --- | --- | --- | --- | --- | --- |
|  | Mirada | | MVision | | | Radformation | | RayStation | | | Therapanacea | | |
|  | vDSC | sDSC | vDSC | | sDSC | vDSC | sDSC | vDSC | | sDSC | vDSC | | sDSC |
| Heart | 0.919 | 0.314 | 0.944 | 0.417 | | 0.947 | 0.424 | 0.935 | 0.400 | | 0.950 | 0.439 | |
| Liver | 0.960 | 0.527 | 0.968 | 0.589 | | 0.966 | 0.565 | 0.961 | 0.539 | | 0.971 | 0.588 | |
| Lung_L | 0.960 | 0.553 | 0.964 | 0.596 | | 0.946 | 0.434 | 0.951 | 0.554 | | 0.963 | 0.585 | |
| Lung_R | 0.965 | 0.531 | 0.971 | 0.601 | | 0.961 | 0.486 | 0.962 | 0.535 | | 0.970 | 0.581 | |
| Oesophagus | 0.539 | 0.373 | 0.798 | 0.572 | | 0.777 | 0.516 | 0.846 | 0.625 | | 0.83 | 0.619 | |
| SpinalCanal | 0.790 | 0.441 | 0.810 | 0.399 | | 0.838 | 0.513 | 0.833 | 0.477 | | 0.842 | 0.543 | |

Supplementary Table 7. Median Hausdorff distance (HD) and median Added Path Length (APL, with no additional tolerance) coefficients for twenty patients and five different AI contouring solutions, for lung models.

| Structure | System | | | | | | | | | | | | |
| --- | --- | --- | --- | --- | --- | --- | --- | --- | --- | --- | --- | --- | --- |
|  | Mirada | | MVision | | | Radformation | | RayStation | | | Therapanacea | | |
|  | HD | APL | HD | | APL | HD | APL | HD | | APL | HD | | APL |
| Heart | 19.5 | 27475 | 11.8 | 23831 | | 14.0 | 25809 | 18.1 | 26167 | | 12.4 | 23937 | |
| Liver | 32.1 | 34201 | 18.8 | 29906 | | 18.6 | 32907 | 26.4 | 32917 | | 16.6 | 30654 | |
| Lung_L | 20.9 | 41129 | 21.3 | 39212 | | 23.2 | 50573 | 30.1 | 42010 | | 21.4 | 41493 | |
| Lung_R | 23.8 | 47344 | 22.9 | 39921 | | 24.4 | 50883 | 31.5 | 50150 | | 26.4 | 42642 | |
| Oesophagus | 86.8 | 5878 | 19.5 | 3619.5 | | 15.4 | 4067 | 12.9 | 2893.5 | | 17.3 | 3551.5 | |
| SpinalCanal | 9.4 | 6145 | 6.1 | 5845.5 | | 5.6 | 5313 | 7.2 | 5270 | | 5.5 | 5631 | |

Supplementary Table 8. Median volumetric Dice (vDSC) and median surface Dice (sDSC, with no additional tolerance) similarity coefficients for twenty patients and five different AI contouring solutions, for prostate models.

| Structure | System | | | | | | | | | | | | |
| --- | --- | --- | --- | --- | --- | --- | --- | --- | --- | --- | --- | --- | --- |
|  | Mirada | | MVision | | | Radformation | | RayStation | | | Therapanacea | | |
|  | vDSC | sDSC | vDSC | | sDSC | vDSC | sDSC | vDSC | | sDSC | vDSC | | sDSC |
| Bladder | 0.947 | 0.618 | 0.970 | 0.739 | | 0.966 | 0.697 | 0.951 | 0.598 | | 0.973 | 0.772 | |
| Bowels | 0.587 | 0.060 | 0.746 | 0.126 | | 0.733 | 0.095 | - | - | | 0.764 | 0.169 | |
| CaudaEquina | - | - | - | - | | 0.753 | 0.393 | - | - | | - | - | |
| FemoralHead_L | 0.906 | 0.614 | 0.907 | 0.663 | | 0.897 | 0.640 | 0.889 | 0.585 | | 0.904 | 0.699 | |
| FemoralHead_R | 0.890 | 0.590 | 0.913 | 0.648 | | 0.905 | 0.615 | 0.883 | 0.509 | | 0.900 | 0.677 | |
| PenileRoot | 0.536 | 0.234 | 0.683 | 0.359 | | 0.663 | 0.352 | - | - | | 0.714 | 0.385 | |
| Prostate | 0.863 | 0.358 | 0.892 | 0.447 | | 0.868 | 0.341 | 0.854 | 0.328 | | 0.905 | 0.482 | |
| Rectum | 0.865 | 0.525 | 0.906 | 0.589 | | 0.883 | 0.572 | 0.870 | 0.528 | | 0.826 | 0.568 | |
| SeminalVesicles | 0.759 | 0.428 | 0.831 | 0.572 | | 0.750 | 0.477 | - | - | | 0.823 | 0.592 | |
| Sigmoid | - | - | 0.770 | 0.551 | | - | - | - | - | | 0.521 | 0.366 | |

Supplementary Table 9. Median Hausdorff distance (HD) and median Added Path Length (APL, with no additional tolerance) coefficients for twenty patients and five different AI contouring solutions, for prostate models.

| Structure | System | | | | | | | | | | | | |
| --- | --- | --- | --- | --- | --- | --- | --- | --- | --- | --- | --- | --- | --- |
|  | Mirada | | MVision | | | Radformation | | RayStation | | | Therapanacea | | |
|  | HD | APL | HD | | APL | HD | APL | HD | | APL | HD | | APL |
| Bladder | 16.2 | 7044 | 6.3 | 5342 | | 6.9 | 6886 | 7.7 | 8203 | | 5.7 | 4753 | |
| Bowels | 75.5 | 1.18E+05 | 76.5 | 1.03E+05 | | 69.2 | 1.13E+05 | - | - | | 55.6 | 1.05E+05 | |
| CaudaEquina | - | - | - | - | | 30 | 3416 | - | - | | - | - | |
| FemoralHead_L | 16.1 | 5586 | 16.9 | 4724 | | 17.2 | 5242 | 25.3 | 5887 | | 18.8 | 4067 | |
| FemoralHead_R | 18.4 | 5624 | 16.0 | 4957 | | 15.9 | 5511 | 27.8 | 6887 | | 18.6 | 4068 | |
| PenileRoot | 11.9 | 794 | 11.0 | 800 | | 11.4 | 758 | - | - | | 8.9 | 698 | |
| Prostate | 7.8 | 3226 | 6.9 | 2957 | | 7.0 | 3591 | 7.8 | 3415 | | 6.9 | 3063 | |
| Rectum | 15.0 | 3802 | 11.6 | 3322 | | 15.6 | 3425 | 19 | 3565 | | 26.0 | 3630 | |
| SeminalVesicles | 9.0 | 1862 | 8.0 | 1489 | | 8.8 | 1817 | - | - | | 7.9 | 1404 | |
| Sigmoid | - | - | 26.3 | 2798 | | - | - | - | - | | 47.1 | 2950 | |
