# Supplementary figures and images for: A clinical evaluation of the performance of five commercial artificial intelligence contouring systems for radiotherapy
Source: Front Oncol. 2023 Aug 4;13:1213068. doi: 10.3389/fonc.2023.1213068 (PMC10436522; doi:10.3389/fonc.2023.1213068)

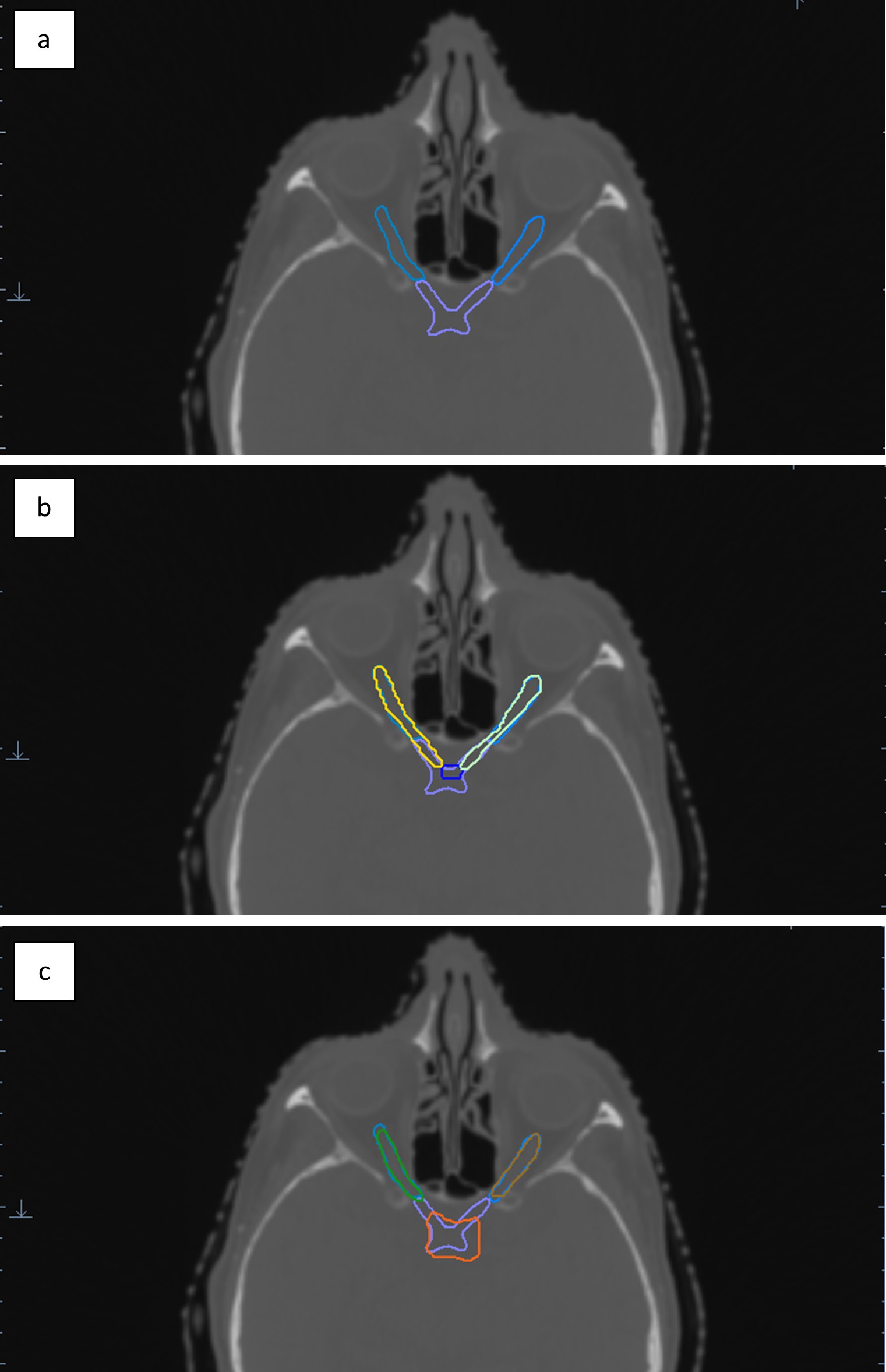

Supplement: Supplementary file 2 [file Image_1.jpeg]

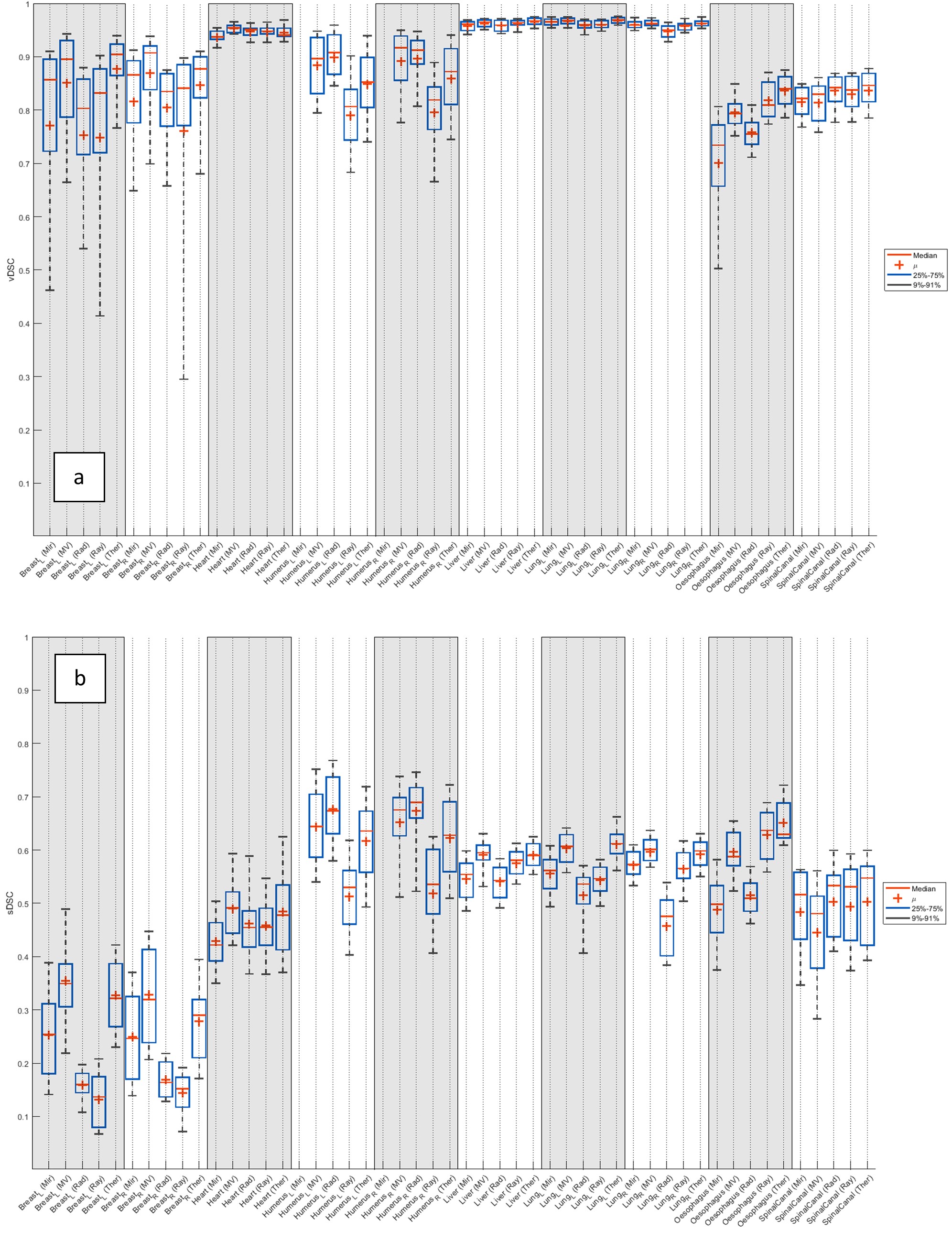

Supplement: Supplementary file 3 [file Image_2.jpeg]

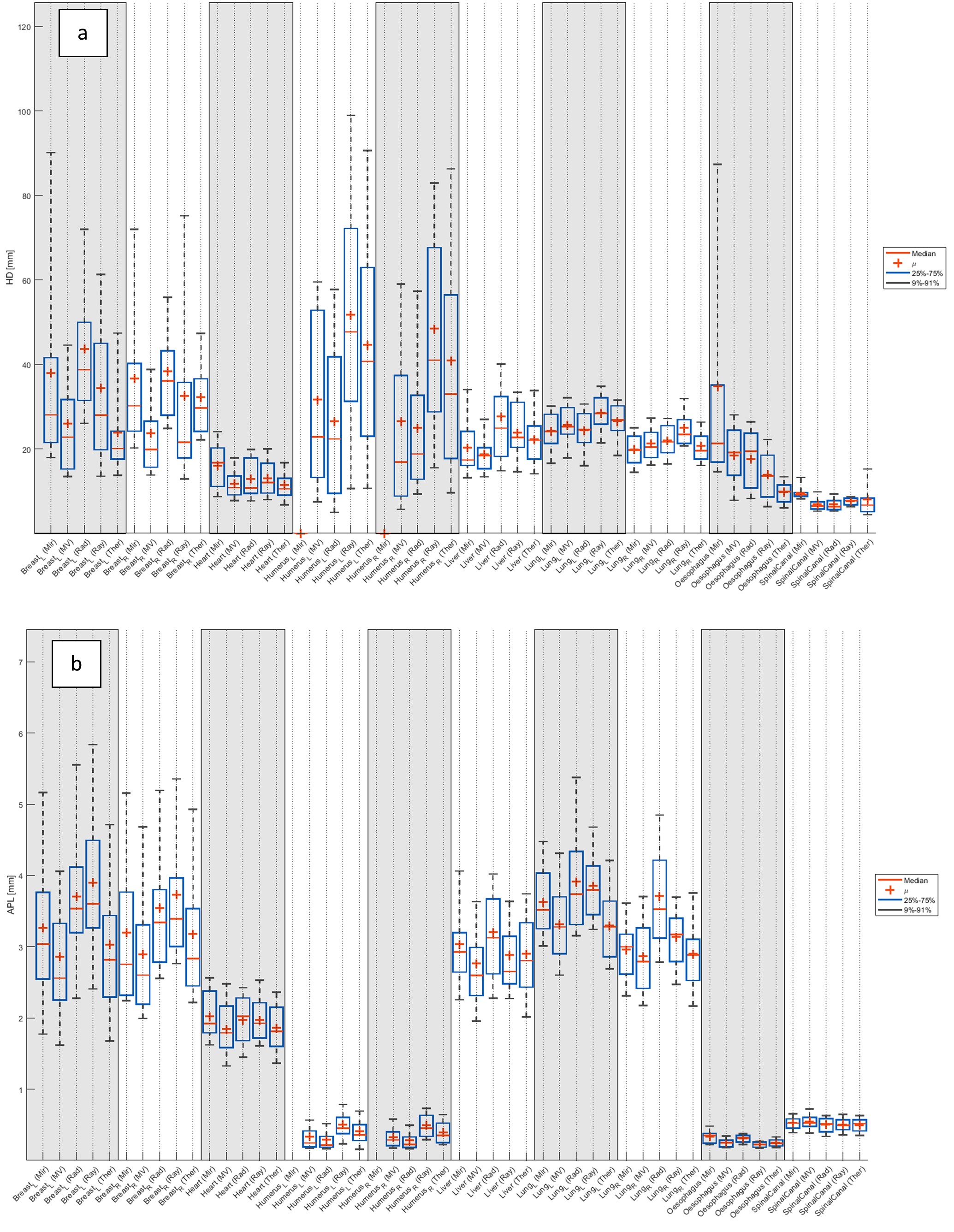

Supplement: Supplementary file 4 [file Image_3.jpeg]

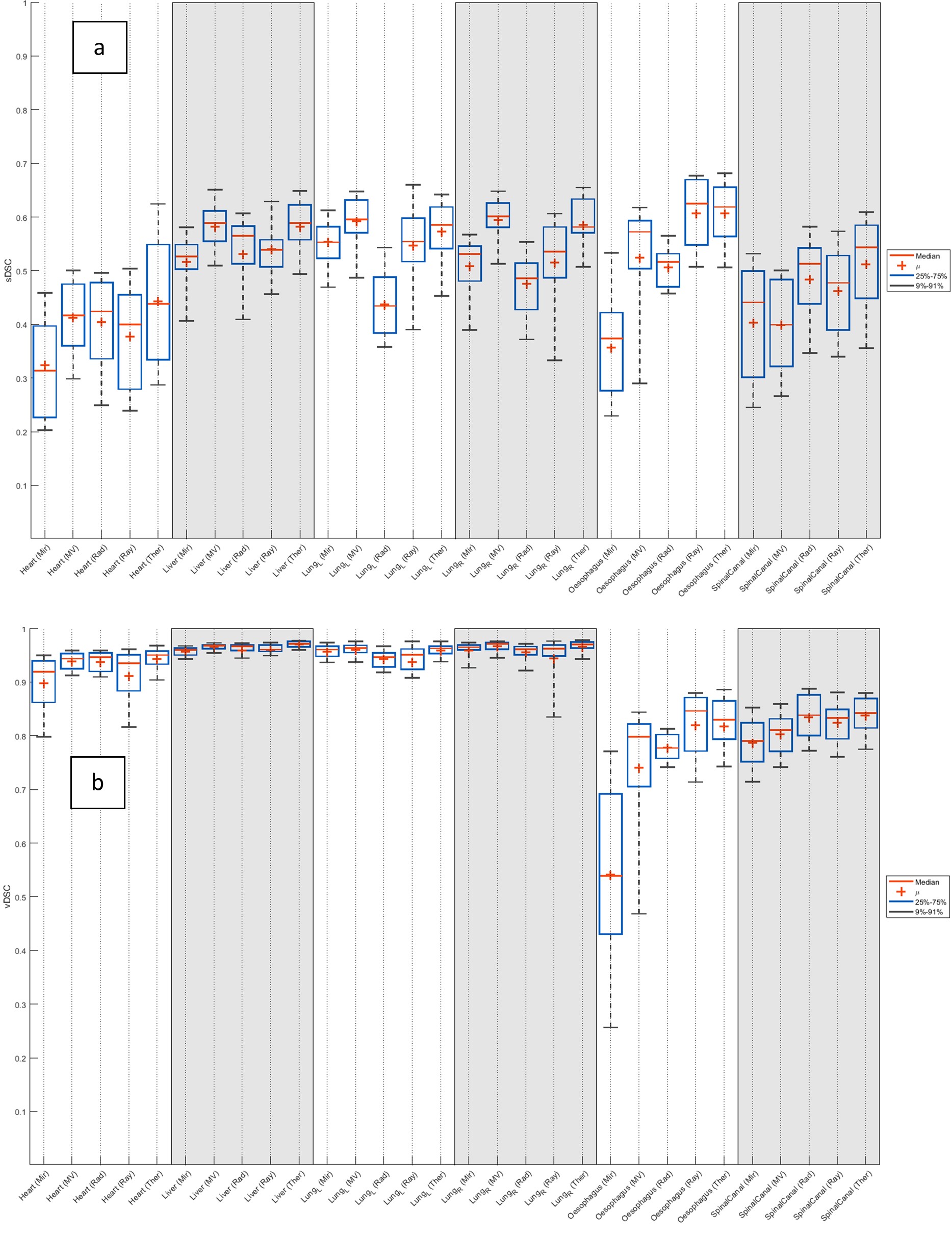

Supplement: Supplementary file 5 [file Image_4.jpeg]

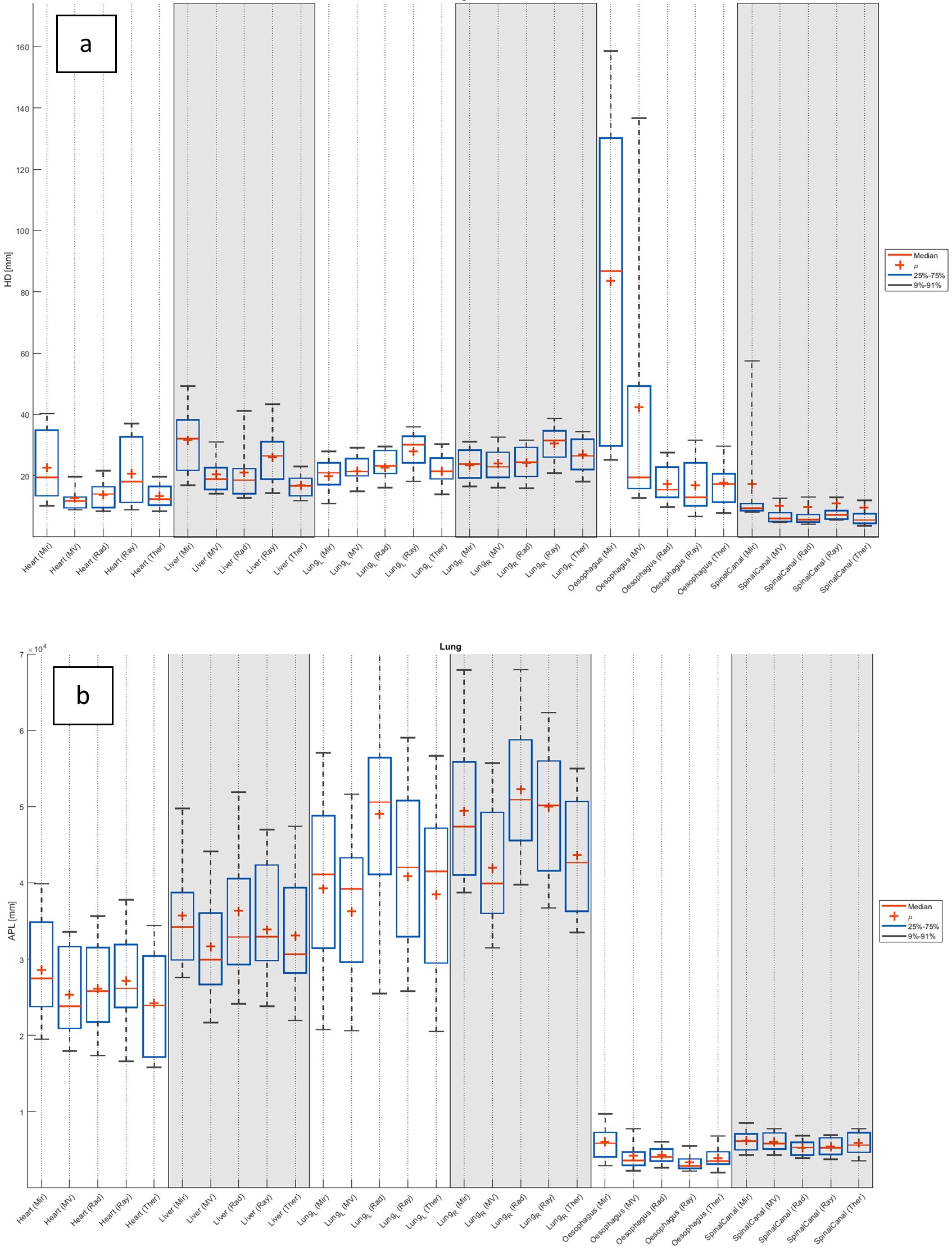

Supplement: Supplementary file 6 [file Image_5.jpeg]

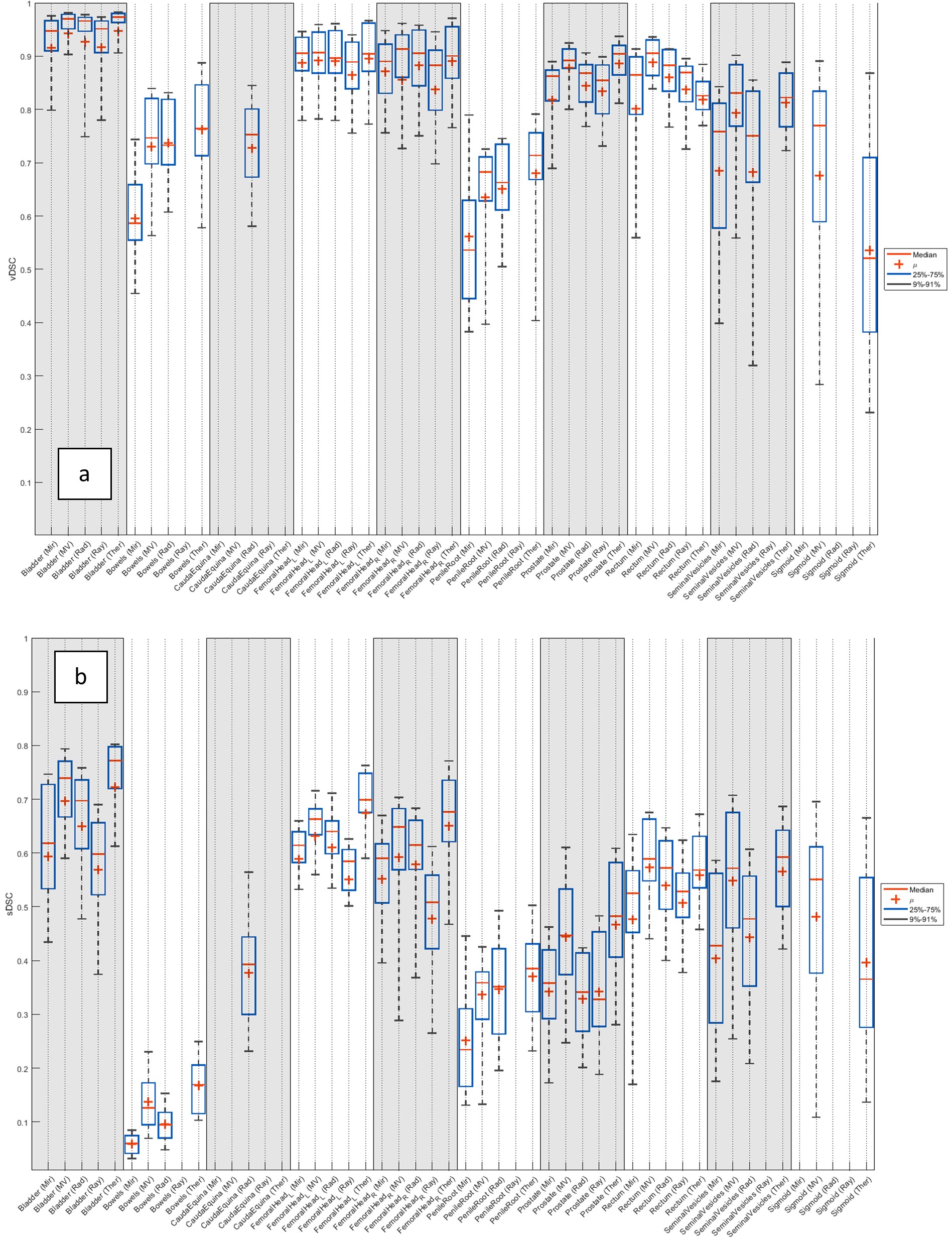

Supplement: Supplementary file 7 [file Image_6.jpeg]

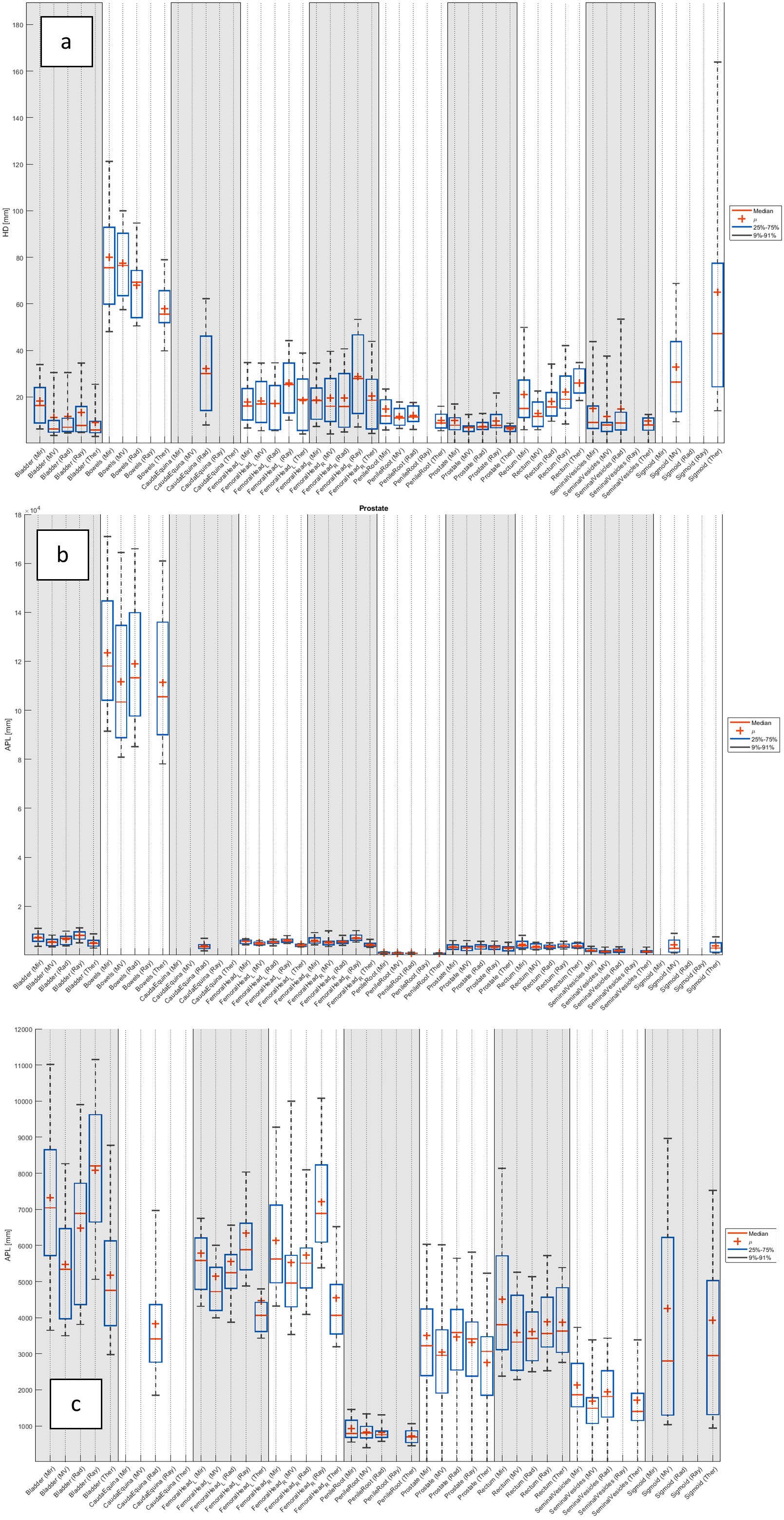

Supplement: Supplementary file 8 [file Image_7.jpeg]
